# Prediction of tumor-reactive T cell receptors from scRNA-seq data for personalized T cell therapy

**DOI:** 10.1038/s41587-024-02161-y

**Published:** 2024-03-07

**Authors:** C. L. Tan, K. Lindner, T. Boschert, Z. Meng, A. Rodriguez Ehrenfried, A. De Roia, G. Haltenhof, A. Faenza, F. Imperatore, L. Bunse, J. M. Lindner, R. P. Harbottle, M. Ratliff, R. Offringa, I. Poschke, M. Platten, E. W. Green

**Affiliations:** 1 CCU Neuroimmunology and Brain Tumor Immunology, German Cancer Research Center, Heidelberg, Germany; 2 German Cancer Consortium, Core Center Heidelberg, Heidelberg, Germany; 3 Department of Neurology, Medical Faculty Mannheim, Mannheim Center for Translational Neuroscience, Heidelberg University, Mannheim, Germany; 4 Faculty of Biosciences, Heidelberg University, Heidelberg, Germany; 5 Immune Monitoring Unit, National Center for Tumor Diseases, Heidelberg, Germany; 6 Helmholtz Institute for Translational Oncology, Mainz, Germany; 7 Department of General, Visceral and Transplantation Surgery, University Hospital Heidelberg, Heidelberg, Germany; 8 Division of Molecular Oncology of Gastrointestinal Tumors, German Cancer Research Center, Heidelberg, Germany; 9 Sino-German Laboratory of Personalized Medicine for Pancreatic Cancer, Union Hospital, Tongji Medical College, Huazhong University of Science and Technology, Wuhan, China; 10 DNA Vector Laboratory, German Cancer Research Center, Heidelberg, Germany; 11 Cellply Srl, Bologna, Italy; 12 BioMed X GmbH, Heidelberg, Germany; 13 Department of Neurosurgery, University Hospital Mannheim, Mannheim, Germany; 14 German Cancer Research Center–Hector Cancer Institute at the Medical Faculty Mannheim, University of Heidelberg, Mannheim, Germany

**Keywords:** Tumour immunology, Tumour immunology

## Abstract

The identification of patient-derived, tumor-reactive T cell receptors (TCRs) as a basis for personalized transgenic T cell therapies remains a time- and cost-intensive endeavor. Current approaches to identify tumor-reactive TCRs analyze tumor mutations to predict T cell activating (neo)antigens and use these to either enrich tumor infiltrating lymphocyte (TIL) cultures or validate individual TCRs for transgenic autologous therapies. Here we combined high-throughput TCR cloning and reactivity validation to train predicTCR, a machine learning classifier that identifies individual tumor-reactive TILs in an antigen-agnostic manner based on single-TIL RNA sequencing. PredicTCR identifies tumor-reactive TCRs in TILs from diverse cancers better than previous gene set enrichment-based approaches, increasing specificity and sensitivity (geometric mean) from 0.38 to 0.74. By predicting tumor-reactive TCRs in a matter of days, TCR clonotypes can be prioritized to accelerate the manufacture of personalized T cell therapies.

## Abstract (web summary)

Tumor-reactive T cell receptors are rapidly identified from lymphocyte scRNA-seq data.

## Main

The success of tumor infiltrating lymphocyte (TIL) therapy trials in metastatic melanoma shows that TILs contain a fraction of tumor-reactive T cells that can be harnessed for adoptive cell therapy^1^. This success is more limited in non-melanoma cancer types^2^ where the baseline fraction of experimentally verifiable, tumor-reactive CD8^+^ T cells is low—often not exceeding 0.5% (ref. ^3^). While the fraction of tumor-reactive T cells can be enriched before reinfusion via cell expansion, this process can exhaust the T cells, compromising their tumor-killing efficacy^4^ and leading to clonal depletion^5^. In contrast, personalized transgenic T cell therapies seek to identify and reinfuse defined tumor-reactive T cell receptors (TCRs), either in patient-autologous T cells^6^ or in induced pluripotent stem cell-derived, hypoimmunogenic (allogeneic) T cells^7^. While this generates a highly efficacious product, identifying tumor-reactive TCRs is a ‘needle in a haystack’ problem^8^. Current techniques place emphasis on tumor antigens, using mutanome analysis to determine the most likely immunogenic neoepitopes to be screened experimentally against TCRs recovered from TILs^9^. This is a technically challenging and time-consuming endeavor: only a fraction of predicted neoepitopes represent physiologically relevant, naturally processed T cell epitopes. Furthermore, while substantial focus has been placed on tumor-specific, single-nucleotide variant (SNV)-derived neoantigens as the source of TCR epitopes, this neglects antigens generated through diverse mechanisms that are only recently beginning to be understood. These include complex mutations such as frame shifts, gene fusions and aberrant gene splicing, as well as novel targets arising though transposable element activation^10^, cell stress-induced tryptophan bumps^11^, aberrant posttranslational modifications^12^, unannotated open reading frames^13^ and even from intracellular pathogens^14^. Together, these ensure that a tumor-focused, antigen-centric approach is both slow and inefficient in identifying suitable tumor-reactive TCRs for use in personalized therapies, thus raising costs and limiting clinical application.

We hypothesized that the identification of tumor-reactive TCRs could be accelerated by developing a TCR-centric, antigen-agnostic approach: ascertaining TCR sequence and tumor reactivity directly from T cells using single-cell combined RNA + VDJ sequencing (scRNA + VDJ-seq). We have previously shown that tumor-infiltrating T cells expressing a TCR against a tumor-specific neoepitope in a vaccinated patient with glioma could be distinguished from bystander T cells on the basis of their expression of *CXCL13* and *CD40LG*^15^. This observation has been extended by other groups using cluster-based differential gene expression analyses to generate multigene ‘signatures’ of tumor-reactive TILs in melanoma^16–18^, lung cancer^19,20^, gastrointestinal cancer^21^, pancreatic ductal adenocarcinoma (PDAC)^22^ and metastatic cancer^23^. The reported gene signatures are only partially overlapping, implying that there may be tumor type-specific transcriptional features in TILs.

We postulated that molecular events in the process of T cell activation upon recognition of a tumor antigen are specific for tumor antigens and independent of tumor type. While differences in published signatures of T cell activation might reflect bona fide differences (for example, as a result of distinct tumor microenvironments), they might also reflect genes playing nonessential roles in T cell activation. In addition to this, the process of validating the tumor reactivity of a TCR requires the generation of tumor models that accurately recapitulate the mutational landscape and epitope processing capacity of the tumor—a process complicated by the spatial heterogeneity of many tumors. The consequence of this is that existing datasets might be noisy due to false negative TCR testing results, in which the tumor model lacks many target epitopes found in the primary tumor. Furthermore, the cost-intensive nature of these experiments and the desire to discover therapeutically useful TCRs has meant that experiments have typically focused on validating TCR clonotypes most likely to be tumor reactive rather than unbiased TCR cloning. This bias may complicate the identification of confounding transcriptional signatures not essential for T cell activation in existing data.

We reasoned that resolving these issues would allow tumor-reactive TILs to be identified regardless of tumor type from single-cell RNA sequencing (scRNA-seq) data alone. Furthermore, by cloning TCRs in an unbiased fashion and including large amounts of negative training data, a machine learning classifier could be trained to identify tumor-reactive TCR clonotypes from scRNA + VDJ-seq data in an automated manner.

## Deep screening identifies tumor-reactive TCR from TILs

**Fig. 1:**
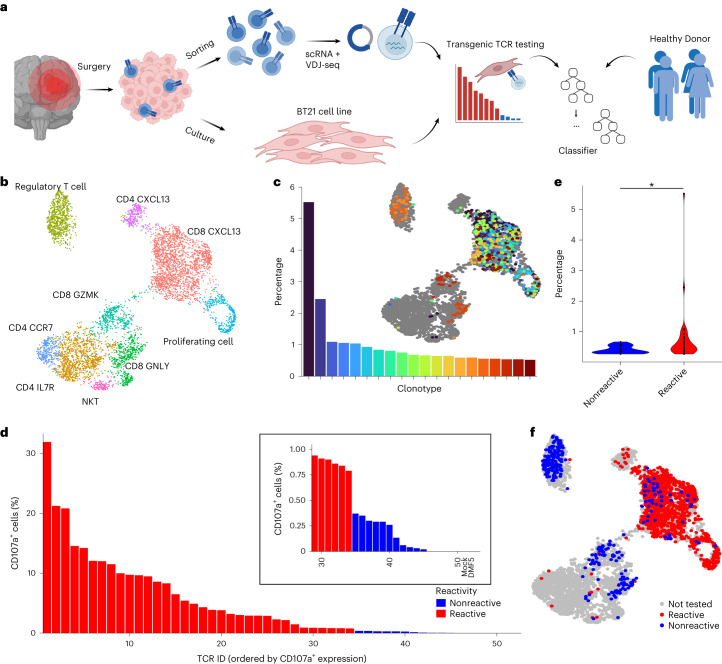
BT21 cell line accurately models resected metastatic lesion, allowing high-confidence experimental TCR tumor-reactivity testing. **a**, An overview of the experimental and computational pipeline underlying the predicTCR classifier: TILs are sorted and subject to scRNA + VDJ-seq, while adjacent resected tumor material is used to establish the BT21 tumor cell line. TCR reactivity data are then integrated with scRNA + VDJ-seq data to train the predicTCR classifier, which is later tested on externally generated TIL datasets from diverse tumor types. **b**, Unsupervised clustering (UMAP plot) of scRNA-seq data of TILs (*n* = 5,651) recovered from brain metastasis sample, with key T cell subtypes annotated. **c**, The percentage frequency of the top 20 TIL TCR clonotypes and their distribution projected onto the UMAP, showing that cells of the same clonotype can occupy diverse phenotypic states. **d**, T cells transfected with one of the 50 most frequently occurring TIL-derived TCR clonotypes (representing 58 distinct TCR α/ß chain pairs) are cocultured with BT21 cells; the resulting levels of CD107a (as quantified by flow cytometry, gated on mTCRβ^+^ cells, which express the transgenic TCR as a chimera with the murine constant domain) demonstrate whether a given TCR clonotype recognizes the BT21 cell line. For details of settings per TCR reactivity threshold, see Methods. DMF5 is the HLA mismatched negative control TCR. **e**, BT21-reactive TCR clonotypes are more frequent than nonreactive clonotypes in the TIL population. **f**, BT21 reactivity testing results projected onto the UMAP plot (**b**). Source data

**Extended Data Fig. 1:**
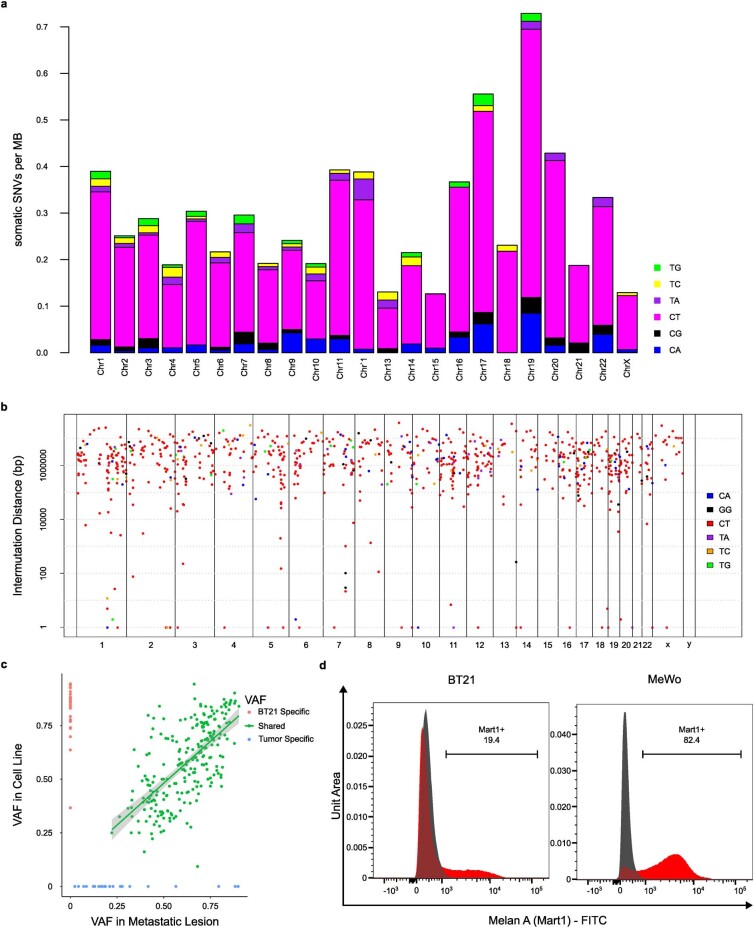
BT21 displays a pattern of mutations characteristic of melanoma. **a**: Metastatic melanoma sample BT21 displays the predominance of UV mediated C > T transition mutations typical of melanoma. **b**: Visualisation of mutations across chromosomes and their density, showing mutations spread throughout the genome, again typical of primary melanoma. **c**: Variant allele frequency (VAF) diagram showing overlap between the SNVs found in BT21 metastatic tumor sample and the BT21 cell line; the cell line’s additional mutations may be the result of tumor heterogeneity or arose during the process of adaptation to cell culture conditions. Full SNV data in *.vcf format are included in Source Data 1. The error bands represent standard error of residuals. **d**: Flow cytometric analysis confirms expression of the tumor associated antigen MART1 in the BT21 cell line as found in 90% of primary cutaneous melanomas^61^. The commonly used MeWo melanoma cell line is shown for comparison. Source data

**Extended Data Fig. 2:**
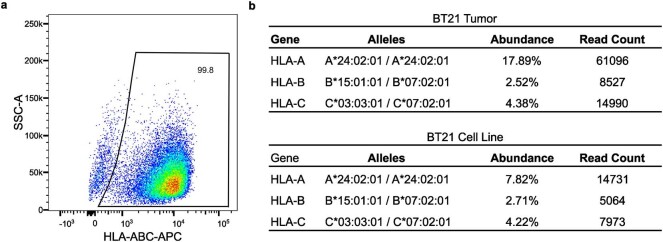
BT21 cell line is suitable for immune testing. **a**: BT21 cell line expresses strong levels of MHC I after two days of stimulation with 300 IU/mL IFNy. **b**: in silico HLA typing using arcasHLA^43^ confirms that the BT21 tumor cell line is derived from primary tumor, and that HLA-A, -B and -C genes are expressed in the absence of stimulation. The ‘Abundance’ metric expresses the percentage of RNAseq reads mapping to HLA alleles, and gives a relative measure of expression between HLA loci. The ‘Read Count’ expresses the absolute number of RNAseq reads mapping to the alleles.

In this study, we set out to identify a tumor sample from which we could sequence TILs and derive a tumor cell line that appropriately recapitulated the primary tumor to allow for high-confidence TCR tumor-reactivity testing to generate a classifier training dataset (Fig. 1a). As sequencing is destructive, adjacent tumor pieces must be used for tumor and TIL sequencing as well as tumor cell line establishment. Tumor mutational heterogeneity (which correlates with TIL heterogeneity^24^) leads to TILs from one tumor piece recognizing antigens absent from the tumor cell line generated from a distal piece, resulting in false negatives during TCR testing that lower the quality of the training dataset. We therefore chose to use a metastatic tumor, as monoclonal metastasis seeding events represent genetic bottlenecks that fix mutations^25^, maximizing the similarity between primary tumor and resultant cell line. We further hypothesized that a metastasis derived from the brain—which has a degree of immune privilege—might result in improved phenotypic separation between bystander and infiltrating tumor-reactive T cells. We identified a metastatic brain tumor from a 62-year-old male patient previously diagnosed with melanoma, which was established as a tumor cell line hereafter termed BT21. Whole-exome sequencing showed that BT21 was a suitable model of the metastatic tumor, sharing 245 of the 268 functional SNVs (Extended Data Fig. 1 and Source data), and constitutively expressing major histocompatibility class I (MHC I) complexes required for epitope presentation and TCR testing (Extended Data Fig. 2).

Unsupervised clustering of scRNA-seq of TIL-derived T cells (*n* = 5,651, hereafter referred to as TILs) showed the presence of distinct clusters expressing known markers of T cell activation including *CXCL13*, *GZMK* and *GNLY* (Fig. 1b). Single-cell VDJ sequencing (scVDJ-seq) of TILs showed the presence of expanded TCR clonotypes, with one clonotype representing over 5% of all clones—a signal indicative of tumor reactivity due to local T cell expansion (Fig. 1c). Additionally, TCR clonotypes found in the scRNA + VDJ of TILs could also be identified in the RNA-seq data derived from a distinct piece of tumor tissue, suggesting that the source tumor was relatively homogeneous in terms of T cell infiltration and presumably the underlying mutational landscape (Supplementary Table 1).

**Extended Data Fig. 3:**
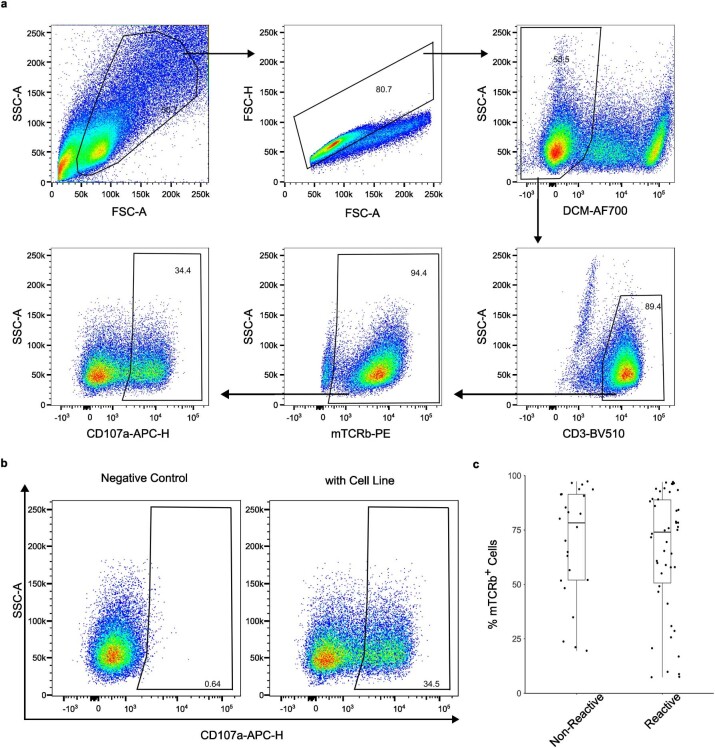
TIL derived TCR testing data using cell line BT21. **a**: Gating strategy for TCR testing. It was gated for lymphocytes, singlet followed by live cells, then CD3^+^ for T Cell, mTCRb^+^ for transgenic T cells then subsequently CD107a for reactivity. **b**: Exemplary flow cytometry data showing the quantification of CD107a of the most reactive BT21 TCR with and without co-culture with BT21 cell line. After co-culture, 34.5% of cells were positive for CD107a. **c**: No significant difference in membrane expression of tumor-reactive and nonreactive TCR clonotypes. The centre line represents median, the lower and upper hinges corresponds to the first and third quantiles, and the upper whisker extends to maxima and lower whisker extends to minima (n = 26 for nonreactive TCR, n = 46 for reactive TCR).

We cloned the most frequently occurring α/ß TCR chain pairs (*n* = 58) from the TIL population (representing 50 distinct TCR clonotypes as some T cells express two productive α chains). TCRs were transfected into expanded healthy donor peripheral blood mononuclear cells (PBMCs) and screened for reactivity against the BT21 cell line using a flow cytometry-mediated readout of T cell activation (CD107a^+^) corrected for per TCR background tonic signaling. A conservative threshold was set to determine TCR reactivity (Methods and Extended Data Fig. 3a,b). We found 34/50 TCRs to be tumor reactive (Fig. 1d and Source data), and showed that there was no significant difference in transgenic TCR expression between reactive and nonreactive TCRs (Extended Data Fig. 3c). Tumor-reactive TCR clonotypes were significantly more expanded in the TIL population than nonreactive clonotypes (Fig. 1e) and individual cells expressing tumor reactive TCRs could occupy various states (Fig. 1b,c,f).

## Development of predicTCR_50_ classifier from scRNA + VDJ data

**Fig. 2:**
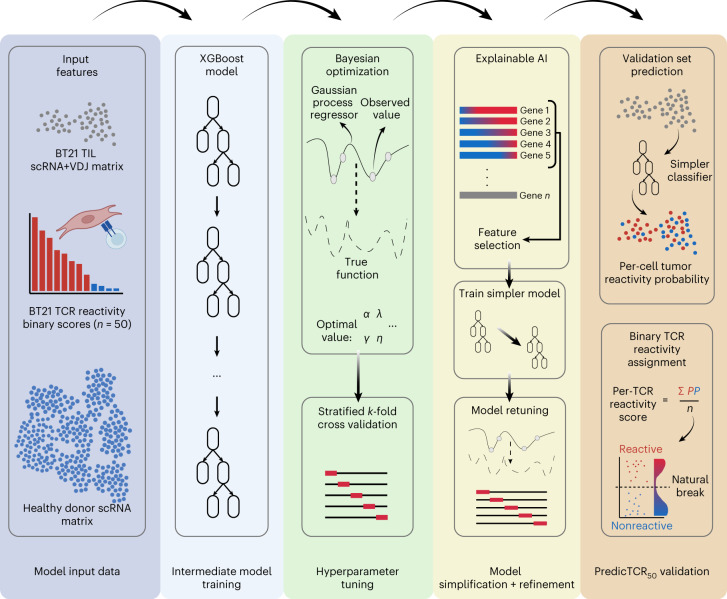
PredicTCR_50_ classifier training strategy. ScRNA data from healthy donors, as well as scRNA + VDJ and experimentally derived tumor-reactivity data for the 50 most frequent TCR TIL clonotypes from sample BT21, were used to train an intermediate model using XGBoost. Due to the sparse nature of scRNA data, we optimized this intermediate model by first performing Bayesian optimization to tune hyperparameters with stratified *k*-fold cross-validation. Subsequently we identified the top features (that is, genes) in this intermediate model using explainable AI SHAP, and then trained a simpler model using only these features to prevent overfitting to the training data. This simpler model was retuned as before and then applied to the remaining BT21 TIL data. Per-cell reactivity probabilities calculated by the classifier were averaged for each TCR clonotype, and the Fisher–Jenk natural break was used to determine the appropriate minimum threshold for calling TCRs as tumor reactive.

**Extended Data Fig. 4:**
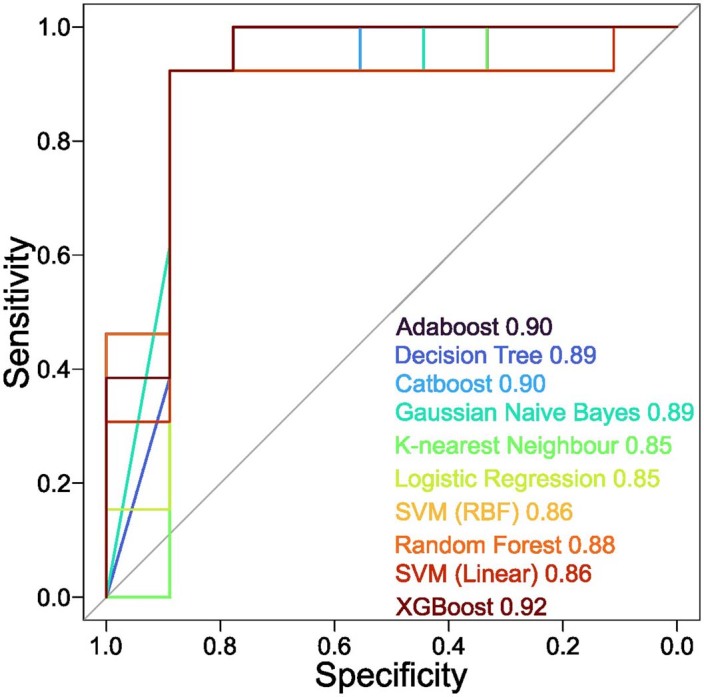
Evaluation of the performance of different machine learning frameworks for TCR tumor-reactivity prediction. All models were implemented using the scikit-learn package, with the exception of XGBoost from xgboost package. The AUC represents model performance without further hyperparameter tuning. Models were trained using the data from 50 BT21 TCRs.

Using the TCR reactivity dataset we established for BT21, we set out to build a machine learning classifier that could accurately and robustly predict tumor reactivity of TIL-derived TCRs based on scRNA + VDJ-seq data using the strategy illustrated in Fig. 2. We first converted the 50 experimentally determined tumor reactivities into a binary label for each TIL cell expressing a tested TCR clonotype and used the corresponding gene expression matrix for those cells as input to train machine learning frameworks. The predictive performance of several machine learning frameworks was evaluated using the area under the receiver operating characteristic (ROC) curve (AUC) metric that ranges between 1 (perfectly predictive), 0.5 (no discrimination capacity between groups) and 0 (reciprocating classes). When making a classifier, a threshold must be set to discriminate between reactive and nonreactive states; the AUC metric assesses the best possible performance of a model on a given dataset by varying the threshold value. This preliminary comparison found eXtreme Gradient Boost (XGBoost)^26^ to be the most suitable framework (Extended Data Fig. 4). While XGBoost performs particularly well due to its ability to update subsequent decision trees during boosting, it has additional advantages for analysis as it effectively implements within-tree parallelization and is able to handle dropout data commonly found in scRNA datasets. Importantly, XGBoost also incorporates regularization to prevent overfitting, which otherwise limits the ability to generalize a model to new datasets.

We added scRNA data from ten healthy donor PBMC samples generated by three independent groups; this produced a maximally diverse negative control dataset for training. Altogether, a total of 112,960 cells were used for training, of which 1,461 cells were TILs from BT21; the imbalanced nature of the training data required careful optimization of data weighting (Methods). XGBoost hyperparameters were tuned using stratified *k*-fold cross-validation with Bayesian optimization, using 70% of the TCRs for training and 30% for testing. To reduce the complexity of our model—important to prevent overfitting that would limit the performance of the classifier on new samples—we identified the key features (that is, genes) determining model performance using explainable artificial intelligence (AI) SHapley Additive exPlanations (SHAP)^27^. We then repeated hyperparameter optimization using only these features.

The probability of tumor reactivity was calculated for each individual T cell using the model, and a mean score then calculated for each TCR clonotype using the data from scVDJ-seq (as TILs expressing a given TCR clonotype may be found occupying various phenotypic states from naive to exhausted; Fig. 1b,c). Finally, the minimum reactivity score required for a TCR clonotype to be called as being tumor reactive was calculated using Fisher–Jenk break optimization, a deterministic statistical analysis that can set sample-specific thresholds. We named the resulting classifier ‘predicTCR_50_’.

## PredicTCR_50_ prediction performance in brain metastasis

**Fig. 3:**
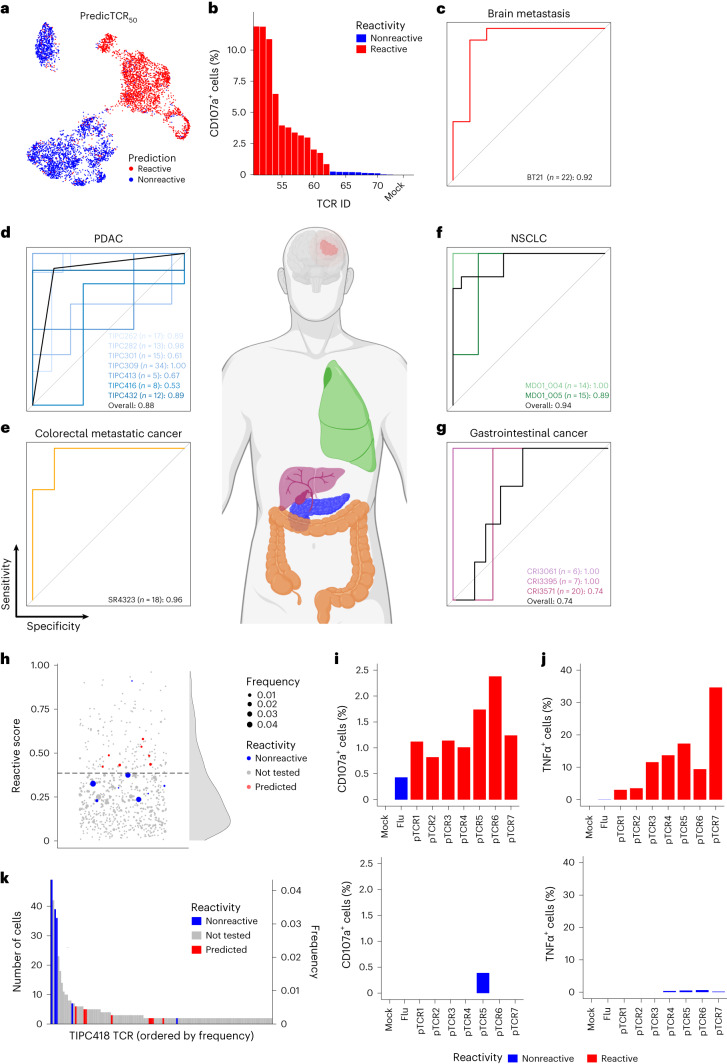
PredicTCR accurately predicts tumor-reactive TCRs in diverse tumor types. **a**, A UMAP plot as in Fig. 1 overlaid with predicTCR_50_ per-cell tumor-reactivity predictions. **b**, An additional 22 TCR clonotypes (29 distinct TCR α/ß chain pairs) were tested for reactivity against the BT21 cell line. **c**, The performance of predicTCR_50_ in prospective prediction of tumor-reactive TCR in BT21 patient. **d**–**g**, The performance of predicTCR in predicting TCR tumor reactivity in published scRNA + VDJ datasets with TCR reactivity data available: seven PDAC samples from Meng et al.^22^ (**d**), one colon metastasis^23^ (**e**), two NSCLC^19^ (**f**) and three gastrointestinal cancers^21^ (**g**). The metrics were calculated by clonotype, with the number of TCR clonotypes for each sample and the AUC value listed. The overall performance was assessed using all available TCRs per cancer modality. Additional metrics and details of the sequencing technology and reactivity testing method used for each sample are listed in Table 3. **h**, PredicTCR reactivity predictions for PDAC sample TIPC418 from Meng et al.^22^ who tested eight TCRs and found none to react to the TIPC418-derived tumor cell line (blue dots, dot size scaled to number of TIL TCR clonotypes). PredicTCR analysis predicted seven of these eight TCRs to be nonreactive (reactivity scores below the Fisher–Jenk natural break threshold, dashed line in plot). Seven additional TCR clonotypes (red dots) predicted to be tumor reactive were cloned for prospective validation of predicTCR. **i**,**j**, Flow cytometry analysis of T cells expressing predicted TIPC418-reactive TCRs cocultured with TIPC418 cells (top) or irrelevant MeWo control cells (bottom) confirmed all seven TCRs to be reactive as assessed by CD107a (**i**) and TNFα (**j**). **k**, The relative frequency and absolute number of recovered TILs for the TCR clonotypes tested in **h**–**j**. Source data

**Extended Data Fig. 5:**
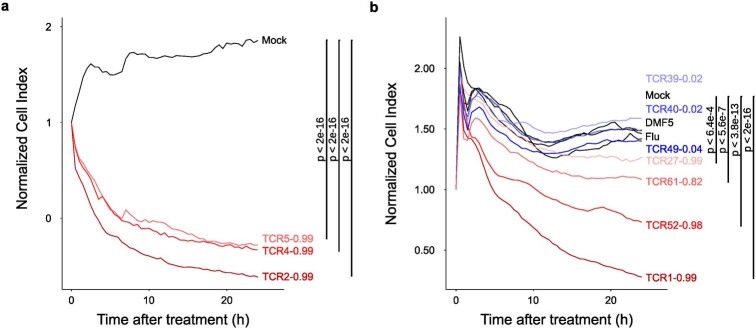
Selected BT21 TIL derived TCRs can kill the BT21 cell line in co-culture assays. **a**: xCELLigence assay showing healthy donor PBMCs transfected with CD8+ TCRs that show high levels of CD107a when co-cultured with BT21 cells (Fig. 1d) can kill BT21 cells in a co-culture assay. **b**: Additional co-culture experiment testing killing capacity of BT21 derived TCRs, including TCRs that predicTCR50 designated as tumor-reactive. Assay was performed using PBMCs from the same healthy donor as in a. Lines are the average of replicates. Statistical analysis was performed as a one-way ANOVA on endpoint normalized cell index with a post-hoc Dunnett test comparison to the Mock. The predicTCR50 score for each TCR clonotype was calculated and added to the plot.

**Extended Data Fig. 6:**
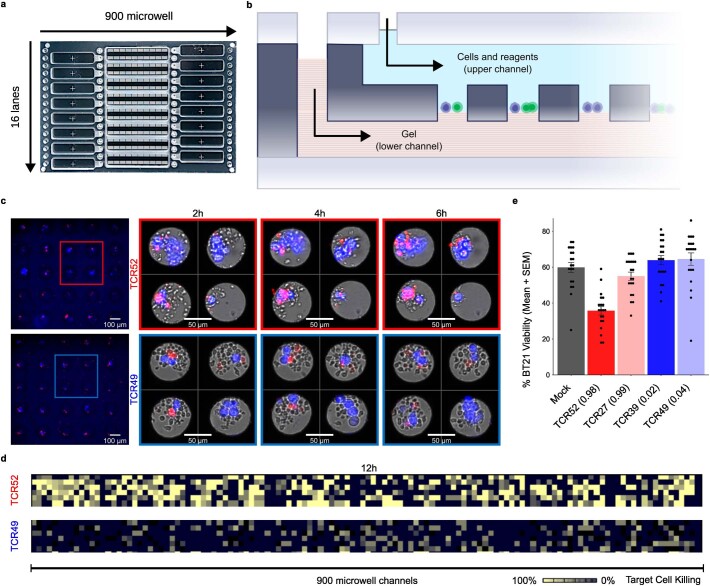
Single cell analysis of killing capacity of TCRs identified by predicTCR. **a**: Micrograph of the CC-Array microfluidic chip measured by the VivaCyteTM platform. **b**: Each lane of the CC-Array device comprises a lower microfluidic channel where the hydrogel is loaded, an upper microfluidic channel where cells and reagents are delivered and an array of microwells opened to both the upper and lower channels. Once the cells are loaded in the upper channel, they settle at the interface with the gel; random loading results in a distribution of effector:tumor cell ratios across each microfluidic channel. **c**: Fluorescence images of a subset of microwells containing a co-culture of PBMCs expressing a transgenic TCR and BT21 target cells at three timepoints (each timepoint shows 4 microwells drawn from 900 replicate microwells). The increase of PI signal (red) on CMA-stained cells (blue) over time reflects killing of the BT21 target cells by the transgenic effector T cells. **d:** Each well is quantified by the VivaCyte software to produce an overview of the killing activity of each TCR. **e**: Quantification of results for four different TCRs recapitulates the xCELLigence data (Extended Data Fig. 5b), with TCR52 performing more efficient target killing than TCR 27 after 12 hours co-culture. predicTCR reactivity scores for each TCR shown in parenthesis. Data are presented as mean values ± SEM for a single experimental run, with each mean representing the average of 20 fields of view (FOV), with each FOV consisting of an average of 45 microwells.

**Extended Data Fig. 7:**
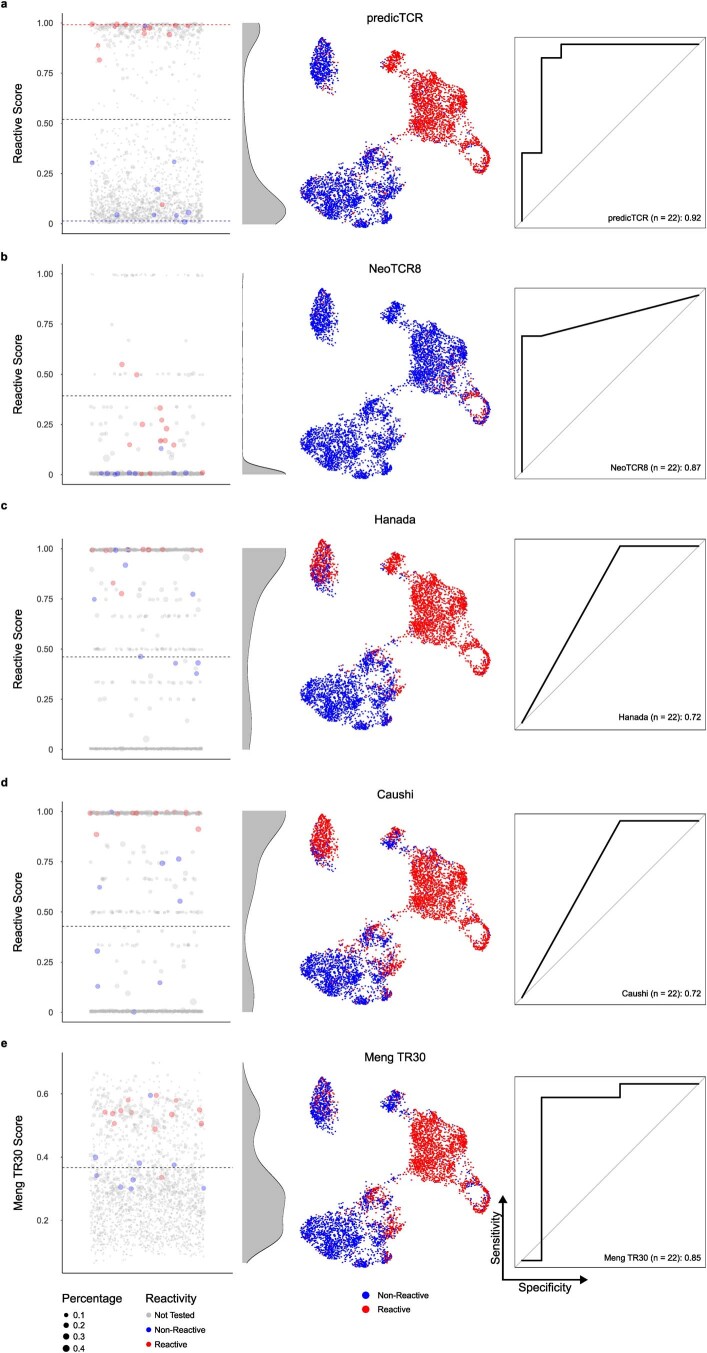
Evaluation of overall performance of gene signature-based TCR tumor-reactivity predictions on BT21 derived TCRs. The left panel in each row shows the distribution of mean tumor-reactivity prediction scores for TIL derived TCR clonotypes from BT21, with a grey shaded density plot on the right. Coloured points show TCR clonotypes in the BT21 validation set (n = 22). TCRs are coloured by experimentally validated reactivity, with the size of each point reflecting the frequency of that TCR clonotype in the TILs. The grey dotted line indicates the sample-specific natural break threshold calculated to separate tumor-reactive from nonreactive clonotypes, while the red and blue dotted lines represent the 95th-percentile and 5th-percentile of tumor-reactivity scores respectively in panel (a). The central panel plots per-cell predictions onto the UMAP plot as in Fig. 1b. The right panel shows the ROC curve and AUC value for each classifier, for details see Table 1. **a**: performance of predicTCR50, showing a clear bimodal distribution of scores that correctly predicts the reactivity of 20 of the 22 validation set TCRs. Note that the false positive prediction (blue dot near top of plot) lies below the 95% threshold line. **b**: Performance of NeoTCR8 from Lowery et al. showing poor prediction of tumor-reactivity for the 22 TCR test set. **c**: Performance of signature developed by Hanada et. al. showing a high false-positive detection rate; note prediction of tumor-reactive T cells in the top left cluster (regulatory T cells, see Fig. 1b). **d**: Performance of signature developed by Caushi et al., also showing a similarly high false positive rate on BT21 TILs. **e**: Performance of signature TR30 from Meng et al. showing higher false positive rate than predicTCR50.

We used predicTCR_50_ to generate tumor-reactivity predictions for all TILs recovered from the BT21 metastasis, with the per-clonotype reactive score clearly separating TCR clonotypes into a bimodal distribution corresponding to reactive and nonreactive TCRs (Fig. 3a). We tested these predictions by cloning and experimentally validating an additional 29 α/ß TCR chain pairs (representing 22 clonotypes; Fig. 3b), finding that predicTCR_50_ accurately predicted tumor reactivity for 20 out of 22 TCRs (AUC of 0.92 and accuracy of 0.91; Fig. 3c and Table 1). Our CD107a^+^ threshold for tumor reactivity captured T cells with a broad range of activated phenotypes, with CD8^+^ BT21-reactive TCRs killing significantly more BT21 cells in bulk culture xCELLigence assays (Extended Data Fig. 5). We were able to recapitulate these results at single-cell resolution by tracking hundreds of individual transgenic effector T cells using miniaturized microwell coculture assays using the Cellply VivaCyte platform (Extended Data Fig. 6). We then implemented the previously published gene signature-based approaches that generate per-cell TCR tumor-reactivity predictions^19,20,23^ and used the same clonotype thresholding procedure as for predicTCR_50_ to distinguish tumor-reactive from nonreactive TCR clonotypes. We found that predicTCR_50_ performed considerably better than the signature-based approaches at predicting reactivity in our 22 TCR set: NeoTCR8 (AUC of 0.87 and accuracy of 0.50), Hanada and Caushi (both AUC of 0.77 and accuracy of 0.72) and Meng TR30 (AUC of 0.85 and accuracy of 0.77) as presented in Table 1 (detailed per-clonotype predictions, Uniform Manifold Approximation and Projection (UMAP) plots and ROC curves shown in Extended Data Fig. 7b–e). This was not unexpected given that the signature approaches were derived from other tumor types, while predicTCR_50_ was trained on BT21 data.

**Table 1:** Performance of tumor-reactivity prediction methods on BT21

Method	Threshold	TP	FP	TN	FN	Accuracy	G-mean	AUC
**NeoTCR8**	0.39	2	0	9	11	0.50	0.15	0.87
**Hanada**	0.43	13	5	4	0	0.82	0.67	0.72
**Caushi**	0.46	13	5	4	0	0.77	0.67	0.72
**Meng TR30**	0.37	12	4	5	1	0.77	0.72	0.85
**PredicTCR**	0.52	12	1	8	1	0.91	0.91	0.92

The performance of each prediction method on the BT21 test set of 22 TCRs was quantified using accuracy, G-mean and AUC. TP, true positive; FP, false positive; TN, true negative; FN, false negative; and threshold, Fisher–Jenk threshold for calling a TCR as tumor reactive.

## Benchmarking predicTCR_50_ false positive rate

**Extended Data Fig. 8:**
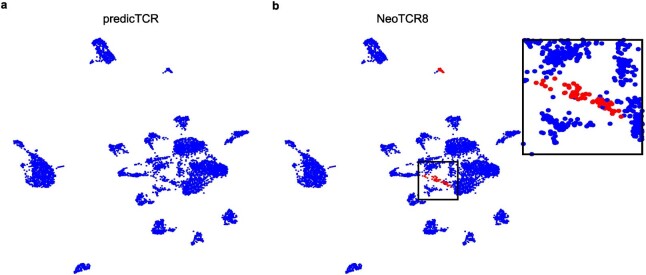
UMAP showing performance of predicTCR and NeoTCR8 on PBMCs from a patient (PP4) with a severely symptomatic COVID-19 infection. **a**: Performance of predicTCR on PBMCs from patient PP4, with no cells predicted to be tumor-reactive. **b**: Performance of NeoTCR8 on same patient; inset panel shows a magnified view of a cluster containing T cells (n = 130, 2% of total cells) called to be tumor-reactive. PBMC data was obtained from Yoshida et al.^28^.

Given that 34/50 of the TCRs in the training set and 13/22 of the TCRs in our validation set were tumor reactive, we questioned whether our predicTCR_50_ classifier might have a bias toward calling TCRs as tumor reactive. Published TCR reactivity datasets (such as those used to derive the signature-based approach) typically present more data for tumor-reactive than nonreactive TCRs; this imbalance means that a classifier that calls many TCRs to be tumor reactive would have an apparently high performance. We therefore evaluated the false positive rate of our classifier by analyzing scRNA data from PBMCs of patients with coronavirus disease 2019 (COVID-19). Severe COVID-19 disease is associated with an enrichment of proliferating and effector memory T_em_ populations^28^, and previous studies have shown that virus-reactive T cells have a transcriptional signature similar to—but distinct from—tumor-reactive T cells. We found that predicTCR_50_ did not classify any T cells from patients with COVID-19 as tumor reactive (Table 2 and Extended Data Fig. 8), suggesting that predicTCR_50_ is highly specific to tumor-reactive T cells and has a low false positive rate. In contrast, gene signature-based approaches such as NeoTCR8 typically called 1–2% of PBMCs as tumor reactive in a majority of patients with COVID-19, even those recovered from infections with mild symptoms where fewer T cells would be expected to express the virus-reactive signature.

**Table 2:** PredicTCR does not falsely detect tumor-reactive T cells in PBMC samples from patients with COVID-19 before and after infection

Severity	No. patients	No. cells	PredicTCR		NeoTCR8	
			PFP	CFP	PFP	CFP
Asymptomatic	5	26,093	0	0	3	260
Mild	6	31,911	0	0	2	95
Moderate	2	19,010	0	0	2	380
Severe	12	74,308	0	0	9	670
Mild – Post	2	11,095	0	0	1	98
Moderate – Post	1	6,865	0	0	0	0
Severe – Post	17	70,087	0	0	4	406

Virus-reactive T cells express a transcriptional program that partially overlaps with that of tumor-reactive T cells. PredicTCR does not call any T cells as being tumor reactive in scRNA + VDJ data from PBMCs drawn from patients with COVID-19 (ref. ^28^), exhibiting better discrimination than gene signature approaches such as the NeoTCR8 signature^23^. Clonotype level analysis not possible as scVDJ-seq was not performed. PFP, the number of patients with false positive cells called as tumor reactive; CFP, the number of cells called as being tumor reactive.

## PredicTCR performance generalizes to diverse tumor types

Having shown that our training method did not generate a classifier with a high false positive rate, we created the final version of predicTCR by retraining on all 72 BT21 derived TCRs (1,679 cells) and healthy donor data (111,499 cells). We set out to compare the performance of predicTCR with that of signature approaches using only externally generated data. Given the aforementioned imbalance in validation data, we primarily used the geometric mean (G-mean) of sensitivity (true positive rate) and specificity (true negative rate) to benchmark model performance. We first applied predicTCR to nine PDAC tumors from which tumor cell lines, TIL scRNA + VDJ data and TCR reactivity testing for 118 clonotypes were available^22^. Despite not being trained on PDAC data, we found that predicTCR could accurately predict experimentally determined tumor reactivity as shown in Fig. 3d and detailed in Table 3 (accuracy of 0.88, G-mean of 0.88 and AUC of 0.88). This suggested that predicTCR detects core transcriptional features of tumor-mediated T cell activation that are independent of tumor type. These scores are notably higher than those achieved when applying gene signature approaches including NeoTCR8 (accuracy of 0.47, G-mean of 0.03 and AUC of 0.65; Supplementary Table 2), Hanada (accuracy of 0.77, G-mean of 0.76 and AUC of 0.76; Supplementary Table 3), Caushi (accuracy of 0.54, G-mean of 0.13 and AUC of 0.51; Supplementary Table 4) and the TR30 signature from Meng et al. (accuracy of 0.81, G-mean of 0.81 and AUC of 0.88; Supplementary Table 5), highlighting the increased predictive value of the machine learning-derived classifier.

**Table 3:** Summary of predicTCR TCR tumor-reactivity predictions in diverse cancer types

Patients	Source	Type	Tech.	Validation	Threshold	TP	FP	TN	FN	Accuracy	G-mean	AUC
TIPC249	Meng et al.	PDAC	10X	Cell line	0.39	5	0	0	1	0.83	NA	NA
TIPC262	Meng et al.	PDAC	10X	Cell line	0.39	9	2	6	0	0.88	0.87	0.89
TIPC282	Meng et al.	PDAC	10X	Cell line	0.39	8	1	4	0	0.92	0.89	0.98
TIPC301	Meng et al.	PDAC	10X	Cell line	0.39	0	1	11	3	0.73	0.00	0.61
TIPC309	Meng et al.	PDAC	10X	Cell line	0.39	21	0	13	0	1.00	1.00	1.00
TIPC413	Meng et al.	PDAC	10X	PDX	0.39	1	0	3	1	0.80	0.71	0.67
TIPC416	Meng et al.	PDAC	10X	Cell line	0.39	3	1	2	2	0.63	0.63	0.53
TIPC418	Meng et al.	PDAC	10X	Cell line	0.39	0	1	7	0	0.88	NA	NA
TIPC432	Meng et al.	PDAC	10X	Cell line	0.39	8	0	3	1	0.92	0.94	0.89
TIPC overall	Meng et al.	PDAC	10X	Cell line	NA	55	6	49	8	0.88	0.88	0.88
SR4323	Lowery et al.	Colon-met	10X	TMG	0.45	11	3	4	0	0.83	0.76	0.96
MD01-004	Caushi et al.	NSCLC	10X	Peptide	0.57	8	2	4	0	0.86	0.82	1.00
MD01-005	Caushi et al.	NSCLC	10X	Peptide	0.45	1	0	12	2	0.87	0.58	0.89
MD043-011	Caushi et al.	NSCLC	10X	Peptide	0.54	2	0	0	0	1.00	NA	NA
MD overall	Caushi et al.	NSCLC	10X	Peptide	NA	11	2	16	2	0.87	0.87	0.94
CRI3061	Zheng et al.	GI (PDAC)	SS2*	TMG + Pep	0.16	2	0	4	0	1.00	1.00	1.00
CRI3244	Zheng et al.	GI (PDAC)	SS2*	TMG + Pep	0.24	0	1	7	0	0.88	NA	NA
CRI3281	Zheng et al.	GI (bile duct)	SS2	TMG + Pep	0.15	0	2	2	0	0.50	NA	NA
CRI3395	Zheng et al.	GI (bile duct)	SS2*	TMG + Pep	0.15	1	1	5	0	0.86	0.91	1.00
CRI3571	Zheng et al.	GI (bile duct)	SS2*	TMG + Pep	0.15	1	14	5	0	0.30	0.51	0.74
CRI overall	Zheng et al.	GI	SS2	TMG + Pep	NA	4	18	23	0	0.60	0.78	0.74

The true positive (TP), false positive (FP), true negative (TN) and false negative (FN) clonotype level tumor-reactivity predictions, as well as the resulting accuracy, G-mean and AUC scores for 18 different tumor samples from four studies. The AUC and G-mean calculations were only possible if both reactive and nonreactive TCRs were available, not available (NA) otherwise. Type, Colon-Met, colorectal metastatic cancer; GI, gastrointestinal cancer. Tech., the single-cell sequencing technology used; 10X, 10X Genomics Single Cell Immune Profiling; SS2, Smart-seq2; SS2*, modified Smart-seq2 (scM&T-Seq); validation, the methodology used to test TCR reactivity; cell line, T cell coculture with tumor cell line, where PDX is T cell coculture with patient-derived xenograft, TMG is T cell coculture with antigen presenter cells expressing a tandem minigene encoding tumor neoantigens and Pep is T cell coculture with peptide-pulsed antigen presenter cells; and threshold, Fisher–Jenk threshold for calling a TCR as tumor reactive.

We next extended our analysis to include additional publicly available TIL scRNA + VDJ datasets from additional tumor types. From Lowery et al., we analyzed a single colorectal metastatic cancer patient (SR4323) for whom both reactive and nonreactive TCRs were available, showing predicTCR accuracy with a G-mean of 0.76 (accuracy of 0.83 and AUC of 0.96; Fig. 3e and Table 3). By comparison, NeoTCR8 performed perfectly on its training dataset with a G-mean of 1.00 (accuracy of 1.00 and AUC of 1.00), whereas the Hanada et al., Caushi et al. and Meng et al. TR30 gene signature-based approaches performed with respective G-means of 0.53 (accuracy of 0.72 and AUC of 0.64), 0.00 (accuracy of 0.61 and AUC of 0.50) and 0.53 (accuracy of 0.72 and AUC of 0.84), suggesting that gene signature-based approaches fail to generalize beyond the tumor type in which they were derived (Supplementary Tables 2–5).

We analyzed three non-small cell lung cancer (NSCLC) samples from Caushi et al. for which TCRs were cloned and tested. Since only ten TCRs were directly tested in these samples, we also included TCRs shown to be neoepitope-reactive based on mutation-associated neoantigen functional expansion or virus-reactive based on viral antigen functional expansion. PredicTCR once again performed well, with a G-mean of 0.87 (accuracy of 0.87 and AUC of 0.94; Fig. 3f and Table 3), better than the Caushi et al. signature derived from these samples, with which we observed a G-mean of 0.75 (accuracy of 0.74 and AUC of 0.83; Supplementary Table 4). The Hanada et al. signature was also derived from NSCLC samples, and as expected, it performed similarly to the Caushi et al. signature with a G-mean of 0.76 (accuracy of 0.81 and AUC of 0.86; Supplementary Table 3). NeoTCR8, on the other hand, was not predictive with a G-mean of 0.00 (accuracy of 0.58 and AUC of 0.50; Supplementary Table 2), while surprisingly the TR30 signature derived from PDAC samples performed well with a G-mean of 0.79 (accuracy of 0.77 and AUC of 0.98).

Finally, we analyzed five gastrointestinal cancer samples generated by Zheng et al.^21^ using Smart-seq2 (ref. ^29^), which contain an order of magnitude fewer cells (mean 328 cells per sample) than other external datasets generated using the 10x Genomics platform^19,20,23^. Notably, this dataset contained testing data for both CD4 and CD8 T cells; however, as published signature-based prediction approaches focused on CD8 cells, we restricted our comparisons to only the CD8 T cell data. For three samples, predicTCR performed with high accuracy (Fig. 3g and Table 3), while for two samples accuracy was reduced, leading to an overall G-mean of 0.78 (accuracy of 0.60 and AUC of 0.74). All other gene signature approaches were not predictive, with G-means of 0.00 (accuracy of 0.91 and AUC of 0.50; Supplementary Table 2), 0.41 (accuracy of 0.24 and AUC of 0.54; Supplementary Table 3), 0.16 (accuracy of 0.11 and AUC of 0.57; Supplementary Table 4) and 0.41 (accuracy of 0.24 and AUC of 0.52; Supplementary Table 5) for NeoTCR8, Hanada, Caushi and Meng TR30 gene signatures, respectively. These results suggest that predicTCR is applicable to datasets with few cells (probably to include tumor biopsies that can be more easily obtained than resection material), and sequenced at lower cost due to the reduced cell number.

Having demonstrated the generalizability of predicTCR, we set out to experimentally validate a number of TCRs predicted to be tumor reactive in a different tumor type. Meng et al. processed a PDAC sample (TIPC418) and tested 12 TCR clonotypes, eight of which were found to be nonreactive and four of which showed weak reactivity^22^. PredicTCR analysis identified many other TCR clonotypes with a high chance of being tumor reactive (Fig. 3h). From these, we selected seven new TCR clonotypes expressed in multiple TILs (Source data) and confirmed that all seven showed reactivity against the TIPC418 PDAC line as assessed by flow cytometry-based quantification of CD107a and TNFα but not against a negative control MeWo cell line (Fig. 3i,j). In this sample, many of the nonreactive TCRs were present at higher frequencies in the TIL population than the reactive clonotypes (Fig. 3k); we found that the two most frequent clonotypes shared a CDR3 α sequence that has been reported to bind to a cytomegalovirus-derived epitope in VDJdb^30^, confirming the utility of predicTCR in identifying TCRs for personalized cell therapies.

## Discussion

Here, we present predicTCR, the first automated classifier of tumor TCR reactivity capable of highly accurate identification of tumor-reactive TCR clonotypes in TILs derived from diverse cancer types through the use of machine learning models combined with deterministic thresholding. We show that through careful sample choice, generation of a large, high-confidence TCR reactivity dataset and inclusion of extensive negative training data, an accurate classifier can be generated. In contrast to previous approaches using differential gene expression to elucidate a gene signature specific to one tumor type, predicTCR enables rapid, antigen-agnostic identification of tumor-reactive TCRs in diverse tumor types—the first step in manufacturing personalized TCR-transgenic T cell cancer therapies.

**Extended Data Fig. 9:**
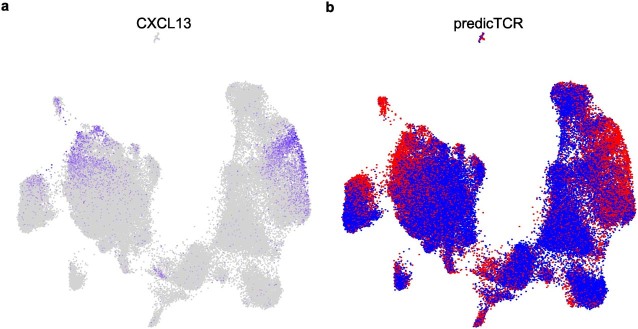
UMAP showing that expression of CXCL13 does not always determine a defined cluster of TILs, but is similar to predicTCR predictions. **a:** T cells expressing CXCL13 in MD01-005 patient do not form a distinct cluster following dimension reduction; this reinforces the difficulty of obtaining accurate results from cluster-based gene set enrichment analyses. **b:** predicTCR tumor-reactivity predictions (which also rely in part on CXCL13) are not limited to a single cluster, and include (but are not limited to) the CXCL13 expressing cells.

The majority of signature-based approaches rely on gene set enrichment analysis of clustered tumor-reactive T cells, and thus identify genes that are upregulated in the *CXCL13* expressing cluster that we have previously shown to contain tumor-reactive infiltrating T cells^15^. However, *CXCL13* expression does not always define a discrete population of T cells (see the example in Extended Data Fig. 9), as cell clustering is highly dependent on upstream processing methods such as normalization^31^, the type of clustering algorithm used and the number of cells in a particular dataset^32^. Furthermore, tumor-reactive TCRs may be expressed in T cells of diverse phenotypes (including memory and exhausted populations): here, cluster-based approaches struggle to interpret genes that are expressed across clusters but which have context-specific predictive value that can be discriminated by machine learning. Finally, clustering approaches require manual verification and annotation to achieve optimal results, making automation difficult. This particularly affects small datasets such as those generated with Smart-seq2, for which cluster-based gene signatures did not detect reactive T cell clusters in as many as six out of ten patients (Zheng et al.^21^).

**Extended Data Fig. 10:**
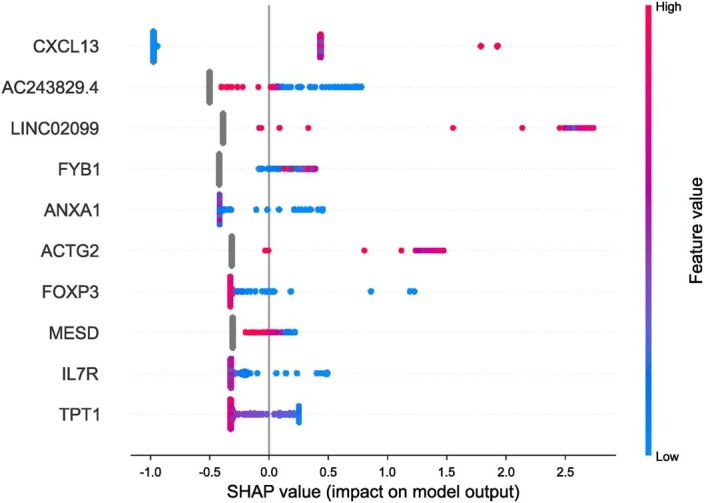
Explainable AI SHAP on predicTCR. SHAP was performed on the predicTCR classifier to identify the top features (genes) that contribute to the classifier. The top 10 genes are illustrated as a beeswarm plot, with each dot representing a single cell. The colour indicates the expression value of a particular gene, and the x-axis indicate the SHAP value, the impact a particular feature on the model output. A positive SHAP value contribute to reactive prediction and vice versa.

We interrogated the predicTCR classifier using explainable AI SHAP to determine the key genes marking tumor reactivity in T cells. While the known reactivity marker *CXCL13* contributed the most to our classifier for prediction (Extended Data Fig. 10), of the two next best genes, AC243829.4 was only identified by Caushi et al., while LINC02099 was completely absent from signature approaches. AC243829.4 has recently been reported to correlate with the presence of immune cells in the tumor microenvironment in clear cell renal cell carcinoma, and is associated with positive patient prognosis^33^, possibly by regulating the expression of the inflammatory cytokine *CCL3* (ref. ^34^). Notably, the relationship between expression of LINC02099 and tumor reactivity is not linear, which we believe is the result of LINC02099 being identified as the hub of a large long noncoding RNA–messenger RNA (mRNA) regulatory network in a breast cancer study^35^, giving rise to complex interaction effects that can be best determined by machine learning approaches.

Given the high cost of manufacturing personalized TCR-mediated cell therapies under GMP or GMP-like conditions, only a limited number of TCRs can be manufactured per patient, so it will be important to avoid manufacturing nontumor-reactive TCRs (that is, false positive predictions). As predicTCR generates per-cell reactivity predictions, predicted reactive TCR clonotypes can be ranked by their mean reactivity score: for BT21 picking TCR clonotypes with reactivity scores above the 95th percentile threshold would exclude the one false positive predicTCR prediction we obtained by using a binary reactivity threshold (Extended Data Fig. 7). Among the TCR clonotypes exceeding a given threshold, the most frequent TCR clonotypes can be prioritized as having a more reliable reactivity score, as well as showing evidence of antigen-driven expansion. Finally, complementary analysis of the CDR3 repertoire can assist in picking between TCRs of similar frequency and score, such as identifying clusters of similar CDR3 sequences that are statistically unlikely to occur in naive repertoires using tools such as ALICE (antigen-specific lymphocyte identification by clustering of expanded sequences)^36^. We illustrate a cluster of TCRs in the BT21 TIL repertoire that have convergently recombined the tumor-reactive CDR3 ß sequence ‘CASSLGGASYEQYF’ in Supplementary Table 6. Of less importance in a translational context is the single false negative reactivity prediction made by predicTCR in the BT21 test set. We speculate that this might reflect a bona fide tumor-reactive TCR which could not be validated using the BT21 cell line, either due to transcriptional changes occurring to the cell line during adaptation to cell culture conditions resulting in downregulation of the TCR’s target antigen or due to the BT21 cell line lacking SNVs found in the original tumor. We note that in general, the performance of predicTCR is better on TCR tumor reactivity datasets generated using a tumor cell line as the T cell target. Tumor cell lines recapitulate the diversity of potential TCR target antigens found in the original tumor, including tumor-associated antigens, posttranslationally modified antigens and neoepitopes derived from cryptic splicing or the dark proteome, some of which are hard to capture using tandem minigene (TMG) assays. It is therefore possible that the higher false positive prediction rate exhibited by predicTCR when analyzing external samples generated using TMG assays to validate TCR reactivity actually reflects a higher false negative TCR reactivity testing rate in the source assays.

In general, predicTCR predicted more tumor-reactive TCR clonotypes for each sample than could be practicably manufactured for a personalized cell therapy. We found this to be the case even for PDAC samples, which are generally considered to be ‘cold tumors’ with a low tumor mutation burden and limited T cell infiltration, which may therefore be refractory to conventional TIL therapies^2^. PredicTCR predictions can be refined with accessory analyses, such as computational prediction of TCR avidity that has been shown to enrich for neoantigen-specific TCRs over tumor-associated antigen-specific TCRs^37^. Optimally, a minimal panel of computationally predicted tumor-reactive TCRs will advance to experimental resolution of the target epitope using sensitive, high dynamic range reporters of T cell activation^38^. Such analyses might be further informed by computational reconstruction of tumor heterogeneity to identify clonal or near-clonal tumor mutations^39^; TCRs reactive to these targets are most likely to result in tumor clearance. Combining these new computational and experimental tools will allow for the creation of a validated patient-derived cell therapy product targeting diverse, tumor-specific, clonal antigens at lower cost than current screening. However, for aggressive cancers in which patient survival is short, the rapid sample-to-vein turnaround enabled by predicTCR would allow for the creation of a personalized cell therapy product in an entirely antigen-agnostic fashion. Although the TCRs contained in such a product would target unknown antigens, given that autologous TCRs have undergone thymic selection, they pose little risk to patients, and targeting subclonal (that is nonoptimal) tumor antigens may yet offer patients clinical benefit by nucleating an immune cascade and epitope spreading effects. Furthermore, resolving the target epitope of a TCR from TCR sequence alone is a rapidly advancing field^40^ and we believe that by pairing tumor mutanome data with predicTCR reactivity predictions, datasets with dramatically reduced numbers of possible TCR–epitope interactions can be generated, serving to train and test TCR–epitope prediction tools, which will themselves allow for the training of TCR reactivity classifiers with ever higher predictive accuracy. As the costs of TCR synthesis fall and validated scRNA + VDJ-seq datasets become more widely available, it will become possible to generate increasingly large training datasets, ensuring that future classifiers can identify tumor-reactive TCRs (or specialized subsets thereof) with even greater accuracy.

In conclusion, we believe that accurate machine learning classifiers such as predicTCR will accelerate the realization of personalized T cell-mediated transgenic cell therapies by reducing overall sample-to-vein turnaround times and increasing the likelihood of therapy delivery before tumor progression, while reducing the costs that currently limit implementation.

## Methods

### Sample and patient consent

Patient BT21, a 62-year-old male previously diagnosed with melanoma, was treated for a brain metastasis at the University Hospital Mannheim following written consent. The patient was not financially compensated for participation. The study was approved by the institutional review board (Ethikkommission 2019-643N).

### Processing of tumor samples for sequencing

Freshly resected brain tumor tissue was obtained from the University Hospital in Mannheim. The patient gave informed written consent before sample collection. Tissue was transported on ice in phosphate-buffered saline (PBS) (Sigma-Aldrich) and processed within 3 h of resection by dissection into into small pieces (2 × 2 × 2 mm). Individual tumor pieces were snap frozen and stored at −80 °C before extracting DNA and RNA for sequencing. The whole-exome library was prepared using SureSelect Human All Exon V7 (5191-4028, Agilent) and the RNA sequencing library was prepared using Ultra Low Input RNA-Seq from TakaraBio. Both were sequenced using NovaSeq 6000 (2× 100 bp). DNA isolated from PBMCs from patient BT21 was included as the whole-exome reference sample.

The remaining tumor pieces were gently mashed through a 100 µm cell strainer using the back side of a syringe plunger to generate a single-cell suspension. To generate a tumor cell line, a portion of the single-cell suspension was spun down (350*g*, 5 min, room temperature) and resuspended in Dulbecco’s modified Eagle medium/F12 (Gibco) supplemented with 1× penicillin–streptomycin (Sigma), 1× B27 supplement (Thermo Fisher), 20 ng ml^−1^ epidermal growth factor (236-EG, R&D Systems) and 20 ng ml^−1^ fibroblast growth factor (13256-029, Thermo Fisher). Cells were placed in a 37 °C CO_2_ incubator where they started to grow as spheroids. Cells were subsequently transferred into Roswell Park Memorial Institute (RPMI)-1640 media (Sigma) supplemented with penicillin–streptomycin and 10% fetal bovine serum (FBS), whereupon they grew as a monolayer. Cells were split with accutase (A1110501, Thermo Fisher) when appropriate during establishment of the robustly growing tumor cell line.

The remaining single-cell suspension was filtered through a 70 µm cell strainer, myelin was removed using myelin removal beads II (130-096-433, Miltenyi) and LS columns (130-042-401, Miltenyi) according to the manufacturer’s protocol, and aliquots of the single-cell suspension were cryopreserved as described for PBMCs. Thawed aliquots were used for fluorescence-activated cell sorting (FACS)-based enrichment of T cells (CD3^+^ and CD45^+^) and prepared for sequencing using Chromium Single Cell V(D)J Reagent kit v1.1 chemistry (PN-1000006, PN-1000020, PN-1000005 and PN-120262, 10X Genomics) according to the manufacturer’s protocol. The constructed scVDJ library and scGEX libraries were sequenced using the NovaSeq 6000 platform (Illumina).

### Exome sequence variant calling

Variant calling was performed by the German Cancer Research Center Omics Data Core Facility using previously described pipelines^41^. Briefly: exome sequencing was performed on DNA extracted from PBMCs, tumor and the tumor cell line. SNVs were called relative to the human genome reference sequence GRCh37, and tumor and cell line SNVs determined by subtracting germline SNVs present in the PBMC sample using the One Touch Pipeline^42^.

### In silico HLA typing from bulk RNA-seq data

For in silico human leukocyte antigen (HLA) typing on paired fastq files from bulk RNA-seq analysis, arcasHLA^43^ was used to perform in silico HLA typing on paired fastq files from bulk RNA-seq analysis.

### Recovery of TCR sequences from bulk RNA-seq data

We used TRUST4 to reconstruct unpaired α and ß TCR chain sequences from within the bulk RNA-seq data as described by Song et al.^44^.

### Generation of TCR in vitro-transcribed mRNA constructs

Cell Ranger-derived TCR clonotype data were processed in R using tidyverse functions^45^. VDJ regions of TCRs were ordered as synthetic DNA fragments from Twist Biosciences and cloned in 96-well format as chimeric TCRs, using murine TRAC or TRBC constant region sequences that had been further modified to include an additional disulfide bond to improve stability and avoid mismatches with the endogenous human TCR after transduction into human T cells^46,47^. As negative controls, we cloned two TCRs targeting HLA-A*02:01 restricted epitopes of MART1 (DMF5 TCR: CDR3α CAVNFGGGKLIF and CDR3β CASSLSFGTEAFF) or influenza (CDR3α CAVSESPFGNEKLTF and CDR3β CASSSTGLPYGYTF). For in vitro transcription, RNA-mediated expression TCR constructs were PCR amplified using a primer to add a T7 promoter, and the resulting PCR product used as a template for the T7 mScript Standard mRNA Production System (CELLSCRIPT C-MSC11610). mRNA was m^7^G capped and enzymatically polyadenylated following the manufacturer’s instructions. For TCR killing assays, TCR constructs were subcloned into S/MAR nanovectors using classical molecular biology techniques as previously described^48^.

### Isolation and expansion of healthy donor PBMCs

PBMCs from healthy donors were isolated from heparinized blood. In short, 15.5 ml of Ficoll Paque Plus Media (Cytiva) was loaded per Leucosep tube (Greiner Bio-One) and spun down. After adding 3 ml of PBS (Sigma), up to 25 ml of blood was loaded on top and a density-gradient centrifugation was performed at 800*g* (acceleration 4 and deceleration 3). After collection of the interphase, PBMCs were washed twice with PBS and frozen in a controlled rate freezing device at −80 °C in 50% freezing medium A (60% X-Vivo 20 and 40% fetal calf serum) and 50% medium B (80% fetal calf serum and 20% dimethylsulfoxide). Cells were stored in liquid nitrogen at −140 °C until further analysis.

The rapid expansion protocol was used to expand T cells. PBMCs from three independent donors were irradiated at 40 Gy using a Gammacell 1000 (AECL) irradiation device to serve as feeder cells. Then, 1 × 10^7^ cells from each donor were pooled together, cells were spun down (400*g*, 10 min, room temperature) and resuspended in rapid expansion protocol media (X-Vivo15 (Lonza, BE02-060Q), 2% human AB serum (H4522-100ML, Sigma-Aldrich), 2.5 µg ml^−1^ Fungizone (15290-018, Gibco), 20 µg ml^−1^ gentamicin (2475.1, Roth), 100 IU ml^−1^ penicillin and 100 µg ml^−1^ streptomycin (15140122, Life Technologies)). Next, 150,000 PBMCs were plated into a standing T25 flask and 666 ng of OKT-3 antibody (Life Technologies, 16-0037-85) was added to the culture and the flask was topped up to a total volume of 20 ml. The next day, 5 ml of X-Vivo15 supplemented with 2% AB serum containing 7,500 IU interleukin-2 (IL-2) was added to the culture. Three days later, 12.5 ml of medium was removed and replaced with 12.5 ml of X-Vivo15 supplemented with 2% AB serum containing 600 IU ml^−1^ IL-2.

### Melan A expression

Melan A expression was confirmed using anti-Melan A-FITC (cat. no. sc-20032, clone A103, Santa Cruz Technology), diluted at 1:10.

### TCR reactivity screening via flow cytometry

TCR-encoding RNA was electroporated into expanded healthy donor PBMCs using the Lonza 4D-Nucleofector (program EO-115, solution P3 supplemented according to the manufacturer’s recommendations), which were plated into 48-well plates containing TexMACS media (130-097-196, Miltenyi) supplemented with 2% human AB serum. At 18–24 h after electroporation, cells were collected and 50 IU ml^−1^ benzonase (YCP1200-50KU, Speed BioSystems) was added to avoid cell clumping. TCR expression levels were measured via flow cytometry with markers including fixable viability dye (AF700, BD), CD3 (clone HIT3A, BV510, BD) and mTCRb (clone H57-597, PE, Biolegend).

To assess TCR reactivity, a total of 150,000 T cells and 75,000 cells of the patient-autologous tumor cell line were cocultured in U-bottom 96-well plates in a total volume of 200 µ. Wells with only T cells, or T cells and TransAct beads (130-111-160, Miltenyi) were used as negative and positive controls, respectively. Then, 5 µl of CD107a FACS antibody (REF 561343, BD) was added per well. After 1 h of coculture, GolgiPlug and GolgiStop (555029 and 554724, BD) were added to reach a 1:1,000 dilution, and after four additional hours of coculture cells were used for flow cytometry analysis. Markers included fixable viability dye (AF700, 1:1,000 dilution, eBioscience), CD3 (clone HIT3A, BV510, 1:20 dilution, BD), mTCRb (clone H57-597, 1:50 dilution, PE, Biolegend) and TNFa (clone MAb11, BV711, 1:10 dilution, Biolegend). Samples were acquired on a FACS Lyric device and flow cytometry data were analyzed using FlowJo software, v10.6.2 (FlowJo LLC).

TCRs were classified as reactive or nonreactive based on flow cytometry data acquired after coculture. The percentage of CD107a positive cells (%CD107a) was quantified by gating on viable CD3^+^ mTCRβ^+^ singlets. TCRs were included in the analysis if the mTCRβ expression was >2%.

The %CD107a signal per TCR after coculture with the cell line (‘TCR versus cell line’) or after running the coculture assay without stimulation (‘TCR, unstimulated’) was corrected for background by calculating

$$\begin{array}{l}( \mathrm\% {\mathrm{CD}}107{{\mathrm{a}}}_\mathrm{TCRvscellline}- \mathrm\% {\mathrm{CD}}107{{\mathrm{a}}}_\mathrm{TCR,unstimulated})-\\( \mathrm\% {\mathrm{CD}}107{{\mathrm{a}}}_\mathrm{Mockvscellline}- \mathrm\% {\mathrm{CD}}107{{\mathrm{a}}}_\mathrm{Mock,unstimulated})\end{array}$$

where mock refers to expanded T cells electroporated without TCR-encoding RNA. TCRs were classified as reactive if the background corrected %CD107a signal per TCR was larger than 2× the standard deviation of the %CD107a^+^ signal measured in all samples without stimulation (1× s.d. of 0.34%). Where a TCR clonotype expressed two α chains, data are presented for the α chain resulting in the %CD107a expression (that is, the functional pair).

### TCR reactivity screening via xCELLigence real-time killing assays

Primary human CD3^+^ cells were isolated from healthy donor volunteers using the Pan T cell isolation kit from Miltenyi Biotec according to the manufacturer’s instructions. The isolated T cells were then activated for 3 days using the human T Cell TransAct kit (Miltenyi Biotec) according to the manufacturer’s instructions and cultured in TexMACS medium from Miltenyi Biotec supplemented with IL-7 and IL-15, both at a final concentration of 10 ng ml^−1^, at a concentration of 1 × 10^6^ cells ml^−1^. 3 days post activation 2 × 10^6^ cells were washed and resuspended in 20 μl of primary P3 solution (Lonza), mixed with 2 μg of S/MAR DNA nanovectors and pulsed with the FI-115 pulsing code using the Lonza 4D-Nucleofector.

Primary human T cells were collected, washed two times and resuspended in FACS buffer (PBS containing 1% of FBS). TCR expression was detected by flow cytometry and T cells were stained with a PE-conjugated antibody (clone H57-597, PE, Biolegend) for 30 min on ice in the dark. Dead cells were excluded by 4,6-diamidino-2-phenylindole gating and alive TCR^+^ cells were gated. Data analysis was performed using FlowJo software.

A real-time killing assay using the xCELLigence was performed. Briefly, BT21 tumor cells were seeded on a 96-well plate (3 × 10^4^ cells per well) and incubated for 24 h. Transgenic T cells were added at an effector–target cell ratio of 2:1 and co-incubated at 37 °C in RPMI 10% medium for 24 h. Cell growth was then monitored for 24 h.

### TCR reactivity screening via cell-mediated cytotoxicity

Analysis of transgenic TCR cell cytotoxicity at microfluidic scale was carried out on the VivaCyte platform (Cellply) loaded with a CC-Array microfluidic device based on a modified version of the open-microwell technology^49^. The CC-Array contains 16 lanes, each lane comprising 1,200 microwells where effector and target cells can interact. Lower microfluidic channels under the microwell array of the CC-Array device were initially preloaded with 6% gelatin methacryloyl hydrogel (900622, Sigma-Aldrich) in PBS and the gel was polymerized with an ultraviolet lamp. BT21 target cells were prestained with CellTracker Blue CMAC Dye (C2110, Thermo Fisher, Invitrogen). Transgenic T cells and BT21 target cells were resuspended in 100% FBS (10270106, Thermo Fisher, Gibco) and loaded on the upper channels of the CC-Array device, resulting in the formation of cocultures on the bottom part of the microwell at the interface between the liquid and the underlying gelatin methacryloyl layer. Each lane was loaded with T cells expressing a single TCR. After cell delivery, a solution of RPMI-1640 (R0883, Sigma-Aldrich) and propidium iodide (P3566, Thermo Fisher) was then delivered into the microchannels and the microfluidic design allowed to rapidly exchange media in the microwells without displacing the cells. The CC-Array device was maintained at 37 °C, 5% CO_2_ and >90% relative humidity in the VivaCyte instrument for the duration of the assay and fluorescence images were acquired every 2 h for 12 h.

An automated analysis of the images was carried out by the VivaCyte software featuring a pretrained deep learning method^50^ to detect target cell cytoplasm. Nine hundred microwells were imaged per microchannel by acquiring 20 subarrays per microchannel. Cell viability was quantified as the frequency of cells stained with CMAC and not stained with propidium iodide.

### ScRNA-seq analysis

Fastq files from sequenced TIL samples were processed using 10X Genomics’ Cellranger v6.1.2 (ref. ^51^) and count matrices are imported into R v4.1 (ref. ^52^). Briefly, SoupX^53^ was used to removed background noise and miQC^54^ used to remove poor quality or degraded cells (that can be identified as having an unusually high mitochondrial gene expression). Cells with an ‘RNA count’ <1,200 and ‘Feature count’ <500 were excluded from further analysis.

### Healthy PBMC datasets

PBMC datasets enriched with T cells from healthy donors were obtained as follows: a single healthy donor PBMC sample from 10X Genomics^55^, two donors from Szabo et al.^56^ and seven donors from Gao et al.^57^. In total, data from 111,499 T cells were obtained.

### PredicTCR classifier training

All scRNA count data from both internally and externally generated datasets were normalized using the ‘sctransform’ method as implemented in Seurat v4 (ref. ^58^), resulting in a gene–cell matrix of Pearson residuals that was used as the model input. TCR reactivity was converted to a binary value from the CD107a flow cytometric quantification as described above; all healthy donor PBMCs were assumed to be nonreactive. The model was trained using scRNA + VDJ-seq data from healthy donors (111,499 cells) plus data from experimentally validated BT21 derived TCRs for predicTCR_50_ (1,461 cells) or predicTCR (1,679 cells) as appropriate. Data were imported in Python (v3.9.16) using pandas (v2.0.2) for preprocessing before training with xgboost (v1.7.4). Due to the scRNA data having many dropouts, we performed hyperparameter tuning before feature selection. The XGBoost hyperparameters ‘colsample_bytree’, ‘gamma’, ‘learning_rate’, ‘max_delta_step’, ‘max_depth’, ‘min_child_weight’, ‘n_estimators’, ‘alpha’, ‘lambda’, ‘scale_pos_weight’ and ‘subsample’ were tuned by Bayesian optimization using scikit-optimize (v0.9.0)^59^ with ten stratified *k*-fold cross-validations to generate an intermediate classifier model. Due to the imbalanced nature of the training dataset, particular attention was put on optimizing data weighting (‘scale_pos_weight’). We used 70% of the data as training data, and the remaining 30% as testing dataset for hyperparameter training. To prevent overfitting to the BT21 training data, we simplified the intermediate classifier using SHAP^27^ to identify the key genes contributing to the model. The final predicTCR classifier was then trained on the top 100 SHAP features and hyperparameters were again optimized as before.

### Prediction of tumor-reactive T cells using predicTCR

External datasets used to validate predicTCR were downloaded and the raw data preprocessed as described above. The prediction probability for each cell was averaged for each clonotype and the subsequent prediction probability for each clonotype was used to calculate the AUC using pROC. The threshold used to classify TCR reactivity was determined using Fisher–Jenk natural break optimization as implemented in jenkspy. The confusion matrix and accuracy of the resulting prediction were then calculated using caret (v6.0-94), and G-mean (the square root of sensitivity and specificity) was calculated using the output of caret.

### Prediction of tumor-reactive T cells using the NeoTCR8 gene signature

Predictions using the NeoTCR8 gene signature were performed as described in Lowery et al. Briefly, the raw gene count matrix was imported into R and scGSEA (using GSVA package, v1.46.0) was performed using the signature gene list (NeoTCR8) obtained from Lowery et al.^23^. Cluster(s) that correspond to 0.95 percentile expression were designated as reactive. A reactive score was calculated using the ratio of predicted reactive cell to the total number of cells for each clonotype. The AUC was then calculated based on this probability score using pROC. To make direct comparisons with the performance of predicTCR, we applied the same Fisher–Jenk optimization to determine the threshold for distinguishing between reactive and nonreactive TCR clonotypes on the basis of the reactive score.

### Prediction of tumor-reactive T cells using the Hanada et al. gene signature

Signature analysis using the Hanada et al. gene signature was performed as described in Hanada et al. Briefly, the raw gene count matrix was imported into R and the score was calculated by adding the genes that contributed positively to the signature and minus the genes that contributed negatively to the signature. Cells that were positive for the signature were called as (neoantigen) reactive. A reactive score was calculated and a minimum threshold for tumor reactivity was determined using Fisher–Jenk optimization as described above.

### Prediction of tumor-reactive T cells using the Caushi et al. gene signature

Signature analysis using the Caushi et al. gene signature was performed similarly to Caushi et al. Briefly, the raw gene count was imported into R and analyzed using Seurat. Seurat was used to normalize the raw count data, then using ‘AddModuleScore’, a signature score was calculated using the mutation-associated neoantigen functional expansion genes. Cells that were positive for the signature were called as reactive. A reactive score was calculated and a minimum threshold for tumor reactivity was determined using Fisher–Jenk optimization as described above.

### Prediction of tumor-reactive T cells using the Meng et al. TR30 gene signature

Signature analysis using Meng TR30 gene signature was performed as described Meng et al.^22^. Briefly, the raw gene count was imported into R and analyzed using Seurat. Seurat was used to normalize the raw count data; then the TR30 signature was computed using the UCell package (v2.2)^60^. The mean of the TR30 signature score was then calculated for each TCR clonotype and termed the Meng TR30 score. The minimum threshold for tumor reactivity was similarly determined using Fisher–Jenk optimization.

### Material availability

The use of the primary tumor cell lines specified in this manuscript is restricted by patient informed consent and institutional review board approval to this study.

### Reporting summary

Further information on research design is available in the Nature Portfolio Reporting Summary linked to this article.

## Online content

Any methods, additional references, Nature Portfolio reporting summaries, source data, extended data, supplementary information, acknowledgements, peer review information; details of author contributions and competing interests; and statements of data and code availability are available at 10.1038/s41587-024-02161-y.

## Supplementary information

Supplementary Information Supplementary Tables 1–6.

Reporting Summary

## Source data

Source Data Fig. 1 Source data for Fig. 1d.

Source Data Fig. 3 Source data for Fig. 3i.

Source Data Extended Data Fig. 1 Source data for Extended Data Fig. 1a.

## Data Availability

Single-cell sequencing data for BT21 have been deposited in National Center for Biotechnology Information BioProject with accession code PRJNA985415. External datasets were obtained from GSE123139, GSE173351 and phs002748.v1.p1. Source data are provided with this paper.

## Code availability

The code is hosted on Zenodo (https://zenodo.org/records/8059129, 10.5281/zenodo.8059129) and will be made available to academic researchers on request.

## References

[1] Rohaan, M. W. et al. Tumor-infiltrating lymphocyte therapy or ipilimumab in advanced melanoma. *N. Engl. J. Med.* **387**, 2113–2125 (2022). 36477031 10.1056/NEJMoa2210233

[2] Monberg, T. J., Borch, T. H., Svane, I. M. & Donia, M. TIL therapy: facts and hopes. *Clin. Cancer Res.* **29**, 3275–3283 (2023). 37058256 10.1158/1078-0432.CCR-22-2428

[3] Cohen, C. J. et al. Isolation of neoantigen-specific T cells from tumor and peripheral lymphocytes. *J. Clin. Invest.* **125**, 3981–3991 (2015). 26389673 10.1172/JCI82416 PMC4607110

[4] Crompton, J. G., Sukumar, M. & Restifo, N. P. Uncoupling T cell expansion from effector differentiation in cell-based immunotherapy. *Immunol. Rev.* **257**, 264–276 (2014). 24329803 10.1111/imr.12135 PMC3915736

[5] Poschke, I. C. et al. The outcome of ex vivo TIL expansion is highly influenced by spatial heterogeneity of the tumor T cell repertoire and differences in intrinsic in vitro growth capacity between T cell clones. *Clin. Cancer Res.* **26**, 4289–4301 (2020). 32303540 10.1158/1078-0432.CCR-19-3845

[6] Zacharakis, N. et al. Immune recognition of somatic mutations leading to complete durable regression in metastatic breast cancer. *Nat. Med.* **24**, 724–730 (2018). 29867227 10.1038/s41591-018-0040-8 PMC6348479

[7] Wang, B. et al. Generation of hypoimmunogenic T cells from genetically engineered allogeneic human induced pluripotent stem cells. *Nat. Biomed. Eng.* **5**, 429–440 (2021). 34002062 10.1038/s41551-021-00730-z

[8] Simoni, Y. et al. Bystander CD8^+^ T cells are abundant and phenotypically distinct in human tumour infiltrates. *Nature* **557**, 575–579 (2018). 29769722 10.1038/s41586-018-0130-2

[9] Hundal, J. et al. PVACtools: a computational toolkit to identify and visualize cancer neoantigens. *Cancer Immunol. Res.* **8**, 409–420 (2020). 31907209 10.1158/2326-6066.CIR-19-0401 PMC7056579

[10] Shah, N. M. et al. Pan-cancer analysis identifies tumor-specific antigens derived from transposable elements. *Nat. Genet.* **55**, 631–639 (2023). 36973455 10.1038/s41588-023-01349-3 PMC13152471

[11] Bartok, O. et al. Anti-tumour immunity induces aberrant peptide presentation in melanoma. *Nature* **590**, 332–337 (2021). 33328638 10.1038/s41586-020-03054-1

[12] Kacen, A. et al. Post-translational modifications reshape the antigenic landscape of the MHC I immunopeptidome in tumors. *Nat. Biotechnol.* **41**, 239–251 (2023). 36203013 10.1038/s41587-022-01464-2 PMC11197725

[13] Ouspenskaia, T. et al. Unannotated proteins expand the MHC-I-restricted immunopeptidome in cancer. *Nat. Biotechnol.* **40**, 209–217 (2022). 34663921 10.1038/s41587-021-01021-3 PMC10198624

[14] Naghavian, R. et al. Microbial peptides activate tumour-infiltrating lymphocytes in glioblastoma. *Nature* **617**, 807–817 (2023). 37198490 10.1038/s41586-023-06081-w PMC10208956

[15] Platten, M. et al. A vaccine targeting mutant IDH1 in newly diagnosed glioma. *Nature* **592**, 463–468 (2021). 33762734 10.1038/s41586-021-03363-z PMC8046668

[16] Oliveira, G. et al. Phenotype, specificity and avidity of antitumour CD8^+^ T cells in melanoma. *Nature* **596**, 119–125 (2021). 34290406 10.1038/s41586-021-03704-y PMC9187974

[17] Oliveira, G. et al. Landscape of helper and regulatory antitumour CD4^+^ T cells in melanoma. *Nature* **605**, 532–538 (2022). 35508657 10.1038/s41586-022-04682-5 PMC9815755

[18] Veatch, J. R. et al. Neoantigen-specific CD4^+^ T cells in human melanoma have diverse differentiation states and correlate with CD8^+^ T cell, macrophage, and B cell function. *Cancer Cell* **40**, 393–409.e9 (2022). 35413271 10.1016/j.ccell.2022.03.006 PMC9011147

[19] Caushi, J. X. et al. Transcriptional programs of neoantigen-specific TIL in anti-PD-1-treated lung cancers. *Nature* **596**, 126–132 (2021). 10.1038/s41586-021-03752-4 PMC8338555 34290408

[20] Hanada, K. et al. A phenotypic signature that identifies neoantigen-reactive T cells in fresh human lung cancers. *Cancer Cell* **40**, 479–493.e6 (2022). 35452604 10.1016/j.ccell.2022.03.012 PMC9196205

[21] Zheng, C. et al. Transcriptomic profiles of neoantigen-reactive T cells in human gastrointestinal cancers. *Cancer Cell* **40**, 410–423.e7 (2022). 35413272 10.1016/j.ccell.2022.03.005

[22] Meng, Z. et al. Transcriptome-based identification of tumor-reactive and bystander CD8^+^ T cell receptor clonotypes in human pancreatic cancer. *Sci. Transl. Med.* **15**, 1–19 (2023). 10.1126/scitranslmed.adh9562 37967201

[23] Lowery, F. J. et al. Molecular signatures of antitumor neoantigen-reactive T cells from metastatic human cancers. *Science* **375**, 877–884 (2022). 35113651 10.1126/science.abl5447 PMC8996692

[24] Joshi, K. et al. Spatial heterogeneity of the T cell receptor repertoire reflects the mutational landscape in lung cancer. *Nat. Med.* **25**, 1549–1559 (2019). 31591606 10.1038/s41591-019-0592-2 PMC6890490

[25] Hu, Z., Li, Z., Ma, Z. & Curtis, C. Multi-cancer analysis of clonality and the timing of systemic spread in paired primary tumors and metastases. *Nat. Genet.* **52**, 701–708 (2020). 32424352 10.1038/s41588-020-0628-z PMC7343625

[26] Chen, T. & Guestrin, C. XGBoost: a scalable tree boosting system. *Proc. 22nd ACM SIGKDD International Conference on Knowledge Discovery and Data Mining* 785–794 (ACM, 2016).

[27] Lundberg, S. M. & Lee, S.-I. in *Advances in Neural Information Processing Systems* Vol. 30 (eds Guyon, I. et al.) (Curran Associates, 2017).

[28] Yoshida, M. et al. Local and systemic responses to SARS-CoV-2 infection in children and adults. *Nature* **602**, 321–327 (2022). 34937051 10.1038/s41586-021-04345-x PMC8828466

[29] Picelli, S. et al. Smart-seq2 for sensitive full-length transcriptome profiling in single cells. *Nat. Methods* **10**, 1096–1100 (2013). 24056875 10.1038/nmeth.2639

[30] Shugay, M. et al. VDJdb: a curated database of T cell receptor sequences with known antigen specificity. *Nucleic Acids Res.* **129**, 170–177 (2017). 10.1093/nar/gkx760 PMC5753233 28977646

[31] Ahlmann-Eltze, C. & Huber, W. Comparison of transformations for single-cell RNA-seq data. *Nat. Methods* **20**, 665–672 (2023). 37037999 10.1038/s41592-023-01814-1 PMC10172138

[32] Yu, L., Cao, Y., Yang, J. Y. H. & Yang, P. Benchmarking clustering algorithms on estimating the number of cell types from single-cell RNA-sequencing data. *Genome Biol.* **23**, 49 (2022). 35135612 10.1186/s13059-022-02622-0 PMC8822786

[33] Wang, Z. et al. Identification and verification of immune subtype-related lncRNAs in clear cell renal cell carcinoma. *Front. Oncol.* **12**, 369–373 (2022). 10.3389/fonc.2022.888502 PMC9200973 35719925

[34] Song, G. Y. et al. Differential expression profiles and functional analysis of long non-coding RNAs in calcific aortic valve disease. *BMC Cardiovasc. Disord.* **23**, 1–13 (2023). 37369992 10.1186/s12872-023-03311-x PMC10294343

[35] Wu, W. et al. Tissue-specific co-expression of long non-coding and coding RNAs associated with breast cancer. *Sci. Rep.* **6**, 1–13 (2016). 27597120 10.1038/srep32731 PMC5011741

[36] Pogorelyy, M. V. et al. Detecting T cell receptors involved in immune responses from single repertoire snapshots. *PLoS Biol.* **17**, 1–13 (2019). 10.1371/journal.pbio.3000314 PMC6592544 31194732

[37] Schmidt, J. et al. Neoantigen-specific CD8 T cells with high structural avidity preferentially reside in and eliminate tumors. *Nat. Commun.* **14**, 3188 (2023). 37280206 10.1038/s41467-023-38946-z PMC10244384

[38] Schmid, T. et al. T-FINDER: a highly sensitive, pan-HLA platform for functional T cell receptor and ligand discovery. *Sci. Adv.* **10**, adk3060 (2024). 10.1126/sciadv.adk3060 PMC10836725 38306432

[39] Grigoriadis, K. et al. CONIPHER: a computational framework for scalable phylogenetic reconstruction with error correction. *Protoc. Exch.* **562**, 833–845 (2023). 10.1038/s41596-023-00913-9 38017136

[40] Jokinen, E. et al. TCRconv: predicting recognition between T cell receptors and epitopes using contextualized motifs. *Bioinformatics* **39**, btac788 (2023). 36477794 10.1093/bioinformatics/btac788 PMC9825763

[41] Campbell, P. J. et al. Pan-cancer analysis of whole genomes. *Nature* **578**, 82–93 (2020). 32025007 10.1038/s41586-020-1969-6 PMC7025898

[42] Reisinger, E. et al. OTP: an automatized system for managing and processing NGS data. *J. Biotechnol.* **261**, 53–62 (2017). 28803971 10.1016/j.jbiotec.2017.08.006

[43] Orenbuch, R. et al. ArcasHLA: high-resolution HLA typing from RNAseq. *Bioinformatics* **36**, 33–40 (2020). 31173059 10.1093/bioinformatics/btz474 PMC6956775

[44] Song, L. et al. TRUST4: immune repertoire reconstruction from bulk and single-cell RNA-seq data. *Nat. Methods* **18**, 627–630 (2021). 33986545 10.1038/s41592-021-01142-2 PMC9328942

[45] Wickham, H. et al. Welcome to the Tidyverse. *J. Open Source Softw.* **4**, 1686 (2019).

[46] Cohen, C. J., Zhao, Y., Zheng, Z., Rosenberg, S. A. & Morgan, R. A. Enhanced antitumor activity of murine–human hybrid T cell receptor (TCR) in human lymphocytes is associated with improved pairing and TCR/CD3 stability. *Cancer Res.* **66**, 8878–8886 (2006). 16951205 10.1158/0008-5472.CAN-06-1450 PMC2147082

[47] Cohen, C. J. et al. Enhanced antitumor activity of T cells engineered to express T cell receptors with a second disulfide bond. *Cancer Res.* **67**, 3898–3903 (2007). 17440104 10.1158/0008-5472.CAN-06-3986 PMC2147081

[48] Bozza, M. et al. A nonviral, nonintegrating DNA nanovector platform for the safe, rapid, and persistent manufacture of recombinant T cells. *Sci. Adv.* **7**, eabf1333 (2021). 33853779 10.1126/sciadv.abf1333 PMC8046366

[49] Bocchi, M. et al. Inverted open microwells for cell trapping, cell aggregate formation and parallel recovery of live cells. *Lab Chip* **12**, 3168–3176 (2012). 10.1039/c2lc40124j 22767321

[50] Schmidt, U., Weigert, M., Broaddus, C. & Myers, G. *Lecture Notes in Computer Science* Vol. 11071 (Springer, 2018).

[51] Zheng, G. X. Y. et al. Massively parallel digital transcriptional profiling of single cells. *Nat. Commun.* **8**, 14049 (2017). 28091601 10.1038/ncomms14049 PMC5241818

[52] *R: A Language and Environment for Statistical Computing* (R Core Team, 2022).

[53] Young, M. D. & Behjati, S. SoupX removes ambient RNA contamination from droplet-based single-cell RNA sequencing data. *Gigascience* **9**, giaa151 (2020). 33367645 10.1093/gigascience/giaa151 PMC7763177

[54] Hippen, A. A. et al. miQC: an adaptive probabilistic framework for quality control of single-cell RNA-sequencing data. *PLoS Comput. Biol.* **17**, e1009290 (2021). 34428202 10.1371/journal.pcbi.1009290 PMC8415599

[55] Human T cells from a healthy donor, 1k cells – multi (v2). *10X Genomics* https://www.10xgenomics.com/datasets/human-t-cells-from-a-healthy-donor-1-k-cells-multi-v-2-2-standard-5-0-0 (2020).

[56] Szabo, P. A. et al. Single-cell transcriptomics of human T cells reveals tissue and activation signatures in health and disease. *Nat. Commun.* **10**, 4706 (2019). 31624246 10.1038/s41467-019-12464-3 PMC6797728

[57] Gao, S. et al. Single-cell RNA sequencing coupled to TCR profiling of large granular lymphocyte leukemia T cells. *Nat. Commun.* **13**, 1982 (2022). 35411048 10.1038/s41467-022-29175-x PMC9001664

[58] Hao, Y. et al. Integrated analysis of multimodal single-cell data. *Cell* **184**, 3573–3587.e29 (2021). 34062119 10.1016/j.cell.2021.04.048 PMC8238499

[59] Head, T. et al. scikit-optimize/scikit-optimize (v0.9.0). *Zenodo*10.5281/zenodo.5565057 (2021).

[60] Andreatta, M. & Carmona, S. J. UCell: robust and scalable single-cell gene signature scoring. *Comput. Struct. Biotechnol. J.* **19**, 3796–3798 (2021). 34285779 10.1016/j.csbj.2021.06.043 PMC8271111

[61] Berset, M. et al. Expression of Melan-A/MART-1 antigen as a prognostic factor in primary cutaneous melanoma. *Int. J. Cancer* **95**, 73–77 (2001). 11241315 10.1002/1097-0215(20010120)95:1<73::aid-ijc1013>3.0.co;2-s

