# Supplementary material for: Prediction of tumor-reactive T cell receptors from scRNA-seq data for personalized T cell therapy
Source: Nat Biotechnol. 2024 Mar 7;43(1):134–42. doi: 10.1038/s41587-024-02161-y (PMC11738991; doi:10.1038/s41587-024-02161-y)
Supplement: Supplementary file 1 — Supplementary Tables 1–6. [file 41587_2024_2161_MOESM1_ESM.pdf]

nature biotechnology

Article

https://doi.org/10.1038/s41587-024-02161-y

Prediction of tumor-reactive T cell receptors from scRNA-seq data for personalized T cell therapy

In the format provided by the authors and unedited

Supplementary Table 1 – BT21 TIL TCR clonotypes are shared between distinct regions of tumor
The BT21 tumor mutanome was determined by bulk RNAseq data performed using RNA isolated from pieces of tumor distinct from that used as the source of TILs for predicTCR scRNA + VDJ-seq analysis and reactivity calling. We used TRUST4[44] to construct rare TCR mRNA sequences present within the bulk RNAseq data, and could show that of the 18 CDR3 sequences recovered, 12 were found in the scRNA + VDJ-seq data, showing considerable overlap in the identity of TILs infiltrating distinct parts of the tumor and confirming the utility of TCR prediction.

CDR3	Bulk RNAseq occurrence count	Chain type	Found in TIL scRNA + VDJ-seq data
CASSFVSGTDTQYF	6	TRB	Yes
CASTRTSVRLGRNEQFF	6	TRB	Yes
CLVGGPYNQGGKLIF	6	NA	NA
CASSFTSGDSPSSYNEQFF	5	TRB	Yes
CASSQVSSYNEQFF	3	TRB	Yes
CASGGSYIPTF	3	NA	NA
CALRGSSNTGKLIF	2	TRA	Yes
CASSLATERGPGETQYF	2	TRB	Yes
CASSFGMRQTQETQYF	2	TRB	Yes
CAIRGDYSYNEQFF	2	TRB	Yes
CAAWELKKLF	2	NA	NA
CAGAWDNNNDMRF	1	TRA	Yes
CAVSNAGNNRKLIW	1	TRA	Yes
CASRPLTGNEQFF	1	TRB	Yes
CASSAGLGRNEQFF	1	TRB	Yes
CASSLVRGSEAFF	1	NA	NA
CLVGDGGGRRALTF	1	NA	NA
CAAQGQAGTALIF	1	NA	NA

Supplementary Table 2 – Table summarising performance of NeoTCR8 gene signature in predicting tumor-reactive TCRs in diverse cancer types.
TP = True Positive, FP = False Positive, TN = True Negative, FN = False Negative, Threshold = Fisher-Jenk threshold for calling a TCR as tumor reactive, NA* = All cells predicted to be nonreactive, no minimum reactivity threshold available.

Patients	Source	Type	Tech	Validation	Threshold	TP	FP	TN	FN	Accuracy	G-Mean	AUC
TIPC249	Meng et al.	PDAC	10X	Cell Line	0.44	0	0	0	6	0.00	NA	NA
TIPC262	Meng et al.	PDAC	10X	Cell Line	0.44	1	0	8	8	0.53	0.33	0.60
TIPC282	Meng et al.	PDAC	10X	Cell Line	0.44	0	0	5	8	0.38	0.00	0.45
TIPC301	Meng et al.	PDAC	10X	Cell Line	0.44	0	0	12	3	0.80	0.00	0.42
TIPC309	Meng et al.	PDAC	10X	Cell Line	0.44	0	0	13	21	0.38	0.00	0.95
TIPC413	Meng et al.	PDAC	10X	PDX	0.44	0	0	3	2	0.60	0.00	0.33
TIPC416	Meng et al.	PDAC	10X	Cell Line	0.44	0	1	2	5	0.25	0.00	0.73
TIPC418	Meng et al.	PDAC	10X	Cell Line	0.44	0	1	7	0	0.88	NA	NA
TIPC432	Meng et al.	PDAC	10X	Cell Line	0.44	1	0	3	8	0.33	0.33	0.56
TIPC Overall	Meng et al.	PDAC	10X	Cell Line	NA	2	2	53	61	0.47	0.03	0.65
SR4323	Lowery et al.	Colon-Met	10X	TMG	0.33	11	0	7	0	1.00	1.00	1.00
MD01-004	Caushi et al.	NSCLC	10X	Peptide	NA*	0	0	6	8	0.43	0.00	0.50
MD01-005	Caushi et al.	NSCLC	10X	Peptide	0.33	0	0	12	3	0.80	0.00	0.50
MD043-011	Caushi et al.	NSCLC	10X	Peptide	NA*	0	0	0	2	0.00	NA	NA
MD Overall	Caushi et al.	NSCLC	10X	Peptide	NA	0	0	18	13	0.58	0.00	0.50
CRI3061	Zheng et al.	GI (PDAC)	SS2*	TMG + Pep	NA*	0	0	4	2	0.67	0.00	0.50
CRI3244	Zheng et al.	GI (PDAC)	SS2*	TMG + Pep	NA*	0	0	8	0	1.00	NA	NA
CRI3281	Zheng et al.	GI (Bile Duct)	SS2	TMG + Pep	NA*	0	0	4	0	1.00	NA	NA
CRI3395	Zheng et al.	GI (Bile Duct)	SS2*	TMG + Pep	NA*	0	0	6	1	0.86	0.00	0.50
CRI3571	Zheng et al.	GI (Bile Duct)	SS2*	TMG + Pep	NA*	0	0	19	1	0.95	0.00	0.50
CRI Overall	Zheng et al.	GI	SS2	TMG + Pep	NA	0	0	41	4	0.91	0.00	0.50

Supplementary Table 3 – Table summarising performance of Hanada gene signature in predicting tumor-reactive TCRs in diverse cancer types.
TP = True Positive, FP = False Positive, TN = True Negative, FN = False Negative, Threshold = Fisher-Jenk threshold for calling a TCR as tumor reactive, NA** = All cells predicted to be reactive, no minimum reactivity threshold available.

Patients	Source	Type	Tech	Validation	Threshold	TP	FP	TN	FN	Accuracy	G-Mean	AUC
TIPC249	Meng et al.	PDAC	10X	Cell Line	0.45	5	0	0	1	0.83	NA	NA
TIPC262	Meng et al.	PDAC	10X	Cell Line	0.45	9	5	3	0	0.71	0.61	0.69
TIPC282	Meng et al.	PDAC	10X	Cell Line	0.45	8	3	2	0	0.77	0.63	0.70
TIPC301	Meng et al.	PDAC	10X	Cell Line	0.45	0	4	8	3	0.53	0.00	0.33
TIPC309	Meng et al.	PDAC	10X	Cell Line	0.45	21	0	13	0	1.00	1.00	1.00
TIPC413	Meng et al.	PDAC	10X	PDX	0.45	2	1	2	0	0.80	0.82	0.83
TIPC416	Meng et al.	PDAC	10X	Cell Line	0.45	2	1	2	3	0.50	0.52	0.53
TIPC418	Meng et al.	PDAC	10X	Cell Line	0.45	0	5	3	0	0.38	NA	NA
TIPC432	Meng et al.	PDAC	10X	Cell Line	0.45	8	0	3	1	0.92	0.94	0.94
TIPC Overall	Meng et al.	PDAC	10X	Cell Line	NA	55	19	36	8	0.77	0.76	0.76
SR4323	Lowery et al.	Colon-Met	10X	TMG	0.33	11	5	2	0	0.72	0.53	0.64
MD01-004	Caushi et al.	NSCLC	10X	Peptide	0.33	5	0	6	3	0.79	0.79	0.81
MD01-005	Caushi et al.	NSCLC	10X	Peptide	0.42	1	1	11	2	0.80	0.55	0.93
MD043-011	Caushi et al.	NSCLC	10X	Peptide	0.45	2	0	0	0	1.00	NA	NA
MD Overall	Caushi et al.	NSCLC	10X	Peptide	NA	8	1	17	5	0.81	0.76	0.86
CRI3061	Zheng et al.	GI (PDAC)	SS2*	TMG + Pep	0.43	2	0	4	0	1.00	1.00	1.00
CRI3244	Zheng et al.	GI (PDAC)	SS2*	TMG + Pep	0.56	0	7	1	0	0.13	NA	NA
CRI3281	Zheng et al.	GI (Bile Duct)	SS2	TMG + Pep	0.50	0	4	0	0	0.00	NA	NA
CRI3395	Zheng et al.	GI (Bile Duct)	SS2*	TMG + Pep	NA**	1	6	0	0	0.14	0.00	0.67
CRI3571	Zheng et al.	GI (Bile Duct)	SS2*	TMG + Pep	0.50	1	17	2	0	0.15	0.32	0.84
CRI Overall	Zheng et al.	GI	SS2	TMG + Pep	NA	4	34	7	0	0.24	0.41	0.54

Supplementary Table 4 – Table summarising performance of Caushi gene signature in predicting tumor-reactive TCRs in diverse cancer types.
TP = True Positive, FP = False Positive, TN = True Negative, FN = False Negative, Threshold = Fisher-Jenk threshold for calling a TCR as tumor reactive, NA** = All cells predicted to be reactive, no minimum reactivity threshold available.

Patients	Source	Type	Tech	Validation	Threshold	TP	FP	TN	FN	Accuracy	G-Mean	AUC
TIPC249	Meng et al.	PDAC	10X	Cell Line	0.50	6	0	0	0	1.00	NA	NA
TIPC262	Meng et al.	PDAC	10X	Cell Line	0.50	9	8	0	0	0.53	0.00	0.50
TIPC282	Meng et al.	PDAC	10X	Cell Line	0.50	8	5	0	0	0.62	0.00	0.50
TIPC301	Meng et al.	PDAC	10X	Cell Line	0.50	3	12	0	0	0.20	0.00	0.50
TIPC309	Meng et al.	PDAC	10X	Cell Line	0.50	21	13	0	0	0.00	0.00	0.50
TIPC413	Meng et al.	PDAC	10X	PDX	0.50	2	2	1	0	0.60	0.58	0.67
TIPC416	Meng et al.	PDAC	10X	Cell Line	0.50	5	3	0	0	0.63	0.00	0.50
TIPC418	Meng et al.	PDAC	10X	Cell Line	0.50	0	8	0	0	0.00	NA	NA
TIPC432	Meng et al.	PDAC	10X	Cell Line	0.50	9	3	0	0	0.75	0.00	0.50
TIPC Overall	Meng et al.	PDAC	10X	Cell Line	NA	63	54	1	0	0.54	0.13	0.51
SR4323	Lowery et al.	Colon-Met	10X	TMG	0.50	11	7	0	0	0.61	0.00	0.50
MD01-004	Caushi et al.	NSCLC	10X	Peptide	0.41	8	5	1	0	0.64	0.41	0.67
MD01-005	Caushi et al.	NSCLC	10X	Peptide	0.53	0	0	12	3	0.80	0.00	0.97
MD043-011	Caushi et al.	NSCLC	10X	Peptide	0.50	2	0	0	0	1.00	NA	NA
MD Overall	Caushi et al.	NSCLC	10X	Peptide	NA	10	5	13	3	0.74	0.75	0.83
CRI3061	Zheng et al.	GI (PDAC)	SS2*	TMG + Pep	0.50	2	4	0	0	0.33	0.00	0.63
CRI3244	Zheng et al.	GI (PDAC)	SS2*	TMG + Pep	0.50	0	8	0	0	0.00	NA	NA
CRI3281	Zheng et al.	GI (Bile Duct)	SS2	TMG + Pep	NA**	0	4	0	0	0.00	NA	NA
CRI3395	Zheng et al.	GI (Bile Duct)	SS2*	TMG + Pep	0.58	1	6	0	0	0.14	0.00	0.50
CRI3571	Zheng et al.	GI (Bile Duct)	SS2*	TMG + Pep	0.75	1	18	1	0	0.10	0.23	0.53
CRI Overall	Zheng et al.	GI	SS2	TMG + Pep	NA	4	40	1	0	0.11	0.16	0.57

Supplementary Table 5 – Table summarising performance of Meng TR30 gene signature in predicting tumor-reactive TCRs in diverse cancer types.
TP = True Positive, FP = False Positive, TN = True Negative, FN = False Negative, Threshold = Fisher-Jenk threshold for calling a TCR as tumor reactive.

Patients	Source	Type	Tech	Validation	Threshold	TP	FP	TN	FN	Accuracy	G-Mean	AUC
TIPC249	Meng et al.	PDAC	10X	Cell Line	0.47	5	0	0	1	0.83	NA	NA
TIPC262	Meng et al.	PDAC	10X	Cell Line	0.47	8	0	8	1	0.94	0.94	0.99
TIPC282	Meng et al.	PDAC	10X	Cell Line	0.47	2	0	5	6	0.54	0.50	0.93
TIPC301	Meng et al.	PDAC	10X	Cell Line	0.47	0	1	11	3	0.73	0.00	0.53
TIPC309	Meng et al.	PDAC	10X	Cell Line	0.47	21	0	13	0	1.00	1.00	1.00
TIPC413	Meng et al.	PDAC	10X	PDX	0.47	1	0	3	1	0.80	0.71	1.00
TIPC416	Meng et al.	PDAC	10X	Cell Line	0.47	1	1	2	4	0.38	0.37	0.47
TIPC418	Meng et al.	PDAC	10X	Cell Line	0.47	0	3	5	0	0.63	NA	NA
TIPC432	Meng et al.	PDAC	10X	Cell Line	0.47	7	0	3	2	0.83	0.88	0.89
TIPC Overall	Meng et al.	PDAC	10X	Cell Line	NA	45	5	50	18	0.81	0.81	0.88
SR4323	Lowery et al.	Colon-Met	10X	TMG	0.22	11	5	2	0	0.72	0.53	0.84
MD01-004	Caushi et al.	NSCLC	10X	Peptide	0.27	8	3	3	0	0.79	0.71	0.94
MD01-005	Caushi et al.	NSCLC	10X	Peptide	0.23	3	4	8	0	0.73	0.82	1.00
MD043-011	Caushi et al.	NSCLC	10X	Peptide	0.33	2	0	0	0	1.00	NA	NA
MD Overall	Caushi et al.	NSCLC	10X	Peptide	NA	13	7	11	0	0.77	0.78	0.98
CRI3061	Zheng et al.	GI (PDAC)	SS2*	TMG + Pep	0.20	2	2	2	0	0.67	0.71	1.00
CRI3244	Zheng et al.	GI (PDAC)	SS2*	TMG + Pep	0.28	0	7	1	0	0.13	NA	NA
CRI3281	Zheng et al.	GI (Bile Duct)	SS2	TMG + Pep	0.28	0	3	1	0	0.25	NA	NA
CRI3395	Zheng et al.	GI (Bile Duct)	SS2*	TMG + Pep	0.28	1	5	1	0	0.29	0.41	0.83
CRI3571	Zheng et al.	GI (Bile Duct)	SS2*	TMG + Pep	0.29	1	17	2	0	0.15	0.32	0.63
CRI Overall	Zheng et al.	GI	SS2	TMG + Pep	NA	4	34	7	0	0.24	0.41	0.52

Supplementary Table 6 – BT21 TILs show evidence of convergent recombination.
Multiple TILs recovered from the BT21 metastatic lesion expressed tumor reactive TCRs with convergently recombined CDR3 sequences.

No. Cells	TRA_CDRD3	TRB_CDR3	TRA_CDR3_NT	TRB_CDR3_NT
21	CAVVNAGNNRKLIW	CASSLGGASYEQYF	TGTGCTGTGGTGAATGCTGGCAACAACCGTAAGCTGATTTGG	TGTGCCAGCAGTTTAGGGGGGGCCTCCTACGAGCAGTACTTC
21	CAVSNAGNNRKLIW	CASSLGGASYEQYF	TGTGCTGTGAGTAATGCTGGCAACAACCGTAAGCTGATTTGG	TGTGCCAGCAGTCTAGGCGGGGCCTCCTACGAGCAGTACTTC
11	CAVSKAGNNRKLIW	CASSLGGASYEQYF	TGTGCTGTGAGTAAAGCTGGCAACAACCGTAAGCTGATTTGG	TGTGCCAGCAGTCTGGGGGGGGCCTCCTACGAGCAGTACTTC
6	CAVVNAGNNRKLIW	CASSLGGASYEQYF	TGTGCTGTCGTTAATGCTGGCAACAACCGTAAGCTGATTTGG	TGTGCCAGCAGTCTCGGGGGTGCCTCCTACGAGCAGTACTTC
3	CAVSNAGNNRKLIW	CASSLGGASYEQYF	TGTGCTGTCTCTAATGCTGGCAACAACCGTAAGCTGATTTGG	TGTGCCAGCAGTTTGGGGGGCGCTTCCTACGAGCAGTACTTC
